# Ungulate Loss and Rewilding Drive Rapid Changes Across Multiple Trophic Levels in an African Savanna

**DOI:** 10.1002/ece3.74703

**Published:** 2026-11-13

**Authors:** Aaron C. Rhodes, Aimee Gaitho, Robert M. Plowes, Colin R. Morrison, Kathryn E. Tisshaw, Amelia A. Wolf, Dino J. Martins, Lawrence E. Gilbert

**Affiliations:** ^1^ Department of Integrative Biology and Brackenridge Field Laboratory The University of Texas at Austin Austin Texas USA; ^2^ Mpala Research Centre Nanyuki Kenya; ^3^ Department of Ecology and Evolution Stony Brook University Stony Brook New York USA

## Abstract

Although the ecological roles of large herbivores in savannas are well studied, the indirect impacts that cascade through community processes remain underexplored, especially during the early stages of reintroducing large herbivores to restore ecological processes or rewilding. In a Kenyan savanna, we used an eight‐year ungulate exclusion experiment with a recent rewilding treatment to test how loss and restoration of ungulate assemblages alter vegetation structure, plant communities, litter, soil conditions, and arthropod communities. Complementing these broader impacts, we used a focal grass species to examine shifts in herbivores and pathogens associated with buffelgrass ( *Cenchrus ciliaris* ), a common palatable C4 perennial bunchgrass used by a wide range of herbivores. Three key patterns emerged from ungulate exclusion and rewilding. First, ungulate exclusion increased buffelgrass volume by 200% (from 2.4 L to 7.5 L m^−2^), but rewilding reversed this effect within 9 months. Second, ungulate exclusion tripled total and pathogen‐associated leaf damage on buffelgrass and increased the number of damage types. Third, ungulate exclusion increased litter and simplified litter arthropod communities, while rewilding increased litter arthropod abundance by 2.4‐fold alongside a partial recovery in litter depth, indicating a rapid detritivore response to rewilding. Additional analyses show treatment‐specific shifts in herbaceous and woody plant communities across precipitation regimes and increased soil compaction with ungulate access. Together, these results show that ungulate exclusion restructures plant communities, increases litter with downstream changes in arthropod communities, and elevates natural enemy pressure on an important native bunchgrass. Meanwhile, rewilding can rapidly reverse many of these biotic effects, even after 8 years of exclusion, though some abiotic legacies persist.

## Graphical abstract

An 8‐year ungulate exclusion and rewilding experiment in Kenyan savannas shows that large herbivores drive both direct and cascading indirect effects on vegetation, soils, arthropods, and pathogens. Ungulate removal increased buffelgrass biomass by 200%, altered plant community structure, and elevated litter accumulation and enemy pressure (including tripled pathogen‐associated damage), while precipitation further modulated these responses. Rewilding rapidly reversed many effects within months, reducing litter, restoring plant structure, and reshaping arthropod and pathogen dynamics, demonstrating strong and rapid ecosystem recovery following herbivore reintroduction.

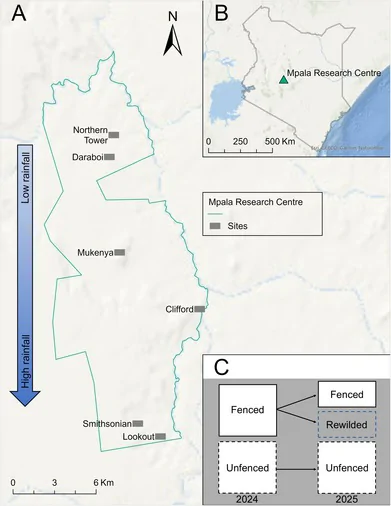

## 1 Introduction

Ungulate abundance and diversity are changing globally and are inextricably linked to anthropogenic activities (Olff et al. 2002; Spear and Chown 2008; Berger et al. 2020). Habitat fragmentation has altered the relationship that ungulates have with the ecosystem on which they depend (Hobbs et al. 2008), and novel processes are emerging as ungulate communities change (Pringle et al. 2007). Ungulates directly influence plant community dynamics and vegetation structure through consumption and indirectly through physical disturbance, ecohydrological processes, and nutrient cycling (Coverdale et al. 2026; Koerner et al. 2018). Through direct plant consumption, ungulate activity can cascade to soil properties and arthropod communities (Ramirez et al. 2021) by altering soil nutrient cycling, compaction, and biota (Sitters and Andriuzzi 2019). Changes in ungulate communities can destabilize community and ecosystem processes in rangelands, and future projections of these alterations are imprecise, at best (Berger et al. 2020). While ungulate declines in many regions have accelerated (di Marco et al. 2014), it is less clear what impact the gain or loss of this important functional group will have on soil and vegetation dynamics, as well as on associated arthropods and pathogens.

To examine these indirect impacts linking ungulate change to other taxa, we focused on buffelgrass ( *Cenchrus ciliaris* ), a common C4 perennial bunchgrass that is highly palatable to a wide range of herbivores and functions as an important species in Kenyan savannas. Buffelgrass is also globally important because it is planted to improve pastureland, yet it increasingly escapes cultivation and becomes a highly invasive grass in arid and semi‐arid ecosystems (Marshall et al. 2012). Its widespread biogeography, palatability, and rich associated communities of arthropods and pathogens make buffelgrass a useful focal species for examining plant and foliar damage responses, as well as broader cascading effects of ungulate loss and restoration on vegetation, litter arthropods, and soil conditions (Morrison et al. 2023; Rhodes et al. 2024). Given that buffelgrass is an important native forage grass and a globally significant invader, understanding how buffelgrass responds to ungulate loss and rewilding has important implications for conservation and rangeland management.

Ungulates can define plant communities by altering the dominance of certain plant species (Koerner et al. 2018) through indirect competition. However, forage selection depends on ungulate size and physiology, allowing them to be categorized as browsers, grazers, or mixed‐feeders (Hanley 1982). Ungulates are top‐down regulators of savanna ecosystems, where the larger megafauna (especially elephants and cape buffalo, *Loxodonta africana* and *Syncerus caffer* , respectively) cause significant physical perturbations to tree and shrub canopies, reducing woody encroachment (Coverdale et al. 2026; Pringle et al. 2023). In comparison, grazers mediate grass‐tree competition, stabilizing the savanna structure of grass‐tree matrices (Donaldson et al. 2022). Size and feeding habits of large ungulates, along with their abundance and community composition, drive plant–plant and plant–animal dynamics across trophic scales (Pringle et al. 2023; Sankaran et al. 2013). Therefore, losing large ungulates can have ecosystem‐shifting impacts at landscape scales (Coverdale et al. 2026). Trophic rewilding reintroduces species to restore top‐down trophic interactions (Svenning et al. 2016) and offers insight into the trophic cascades that losing such species may trigger.

Ungulates alter other arthropod herbivores and pathogens by consuming the plants they rely on, reducing their habitat (where they feed, reproduce, etc.), or by directly ingesting them. As a result, browsing and grazing can reduce arthropod diversity by affecting overall plant biomass and structure (van Klink et al. 2015). However, the impact may be more nuanced, given that ungulate herbivory can reduce specialist insects by damaging host plants and through incidental consumption (Gish et al. 2017), but not affect arthropods with greater mobility (Rhodes et al. 2024). Furthermore, the shifts in vegetation traits and microclimate caused by grazing may benefit some arthropods more than others and are mediated by local water resources (Torma et al. 2023). Similarly, ungulates can directly alter pathogen damage by acting as vectors or by creating entry wounds for pathogens (Emer et al. 2024). Conversely, pathogens may be more dependent on broader climate and weather patterns, and pathogen incidence may be context‐dependent (Rhodes et al. 2024). Ungulates' impact on arthropod communities depends on arthropod functional groups and may differ between living plants and detritus (Daskin and Pringle 2016).

Litter is a cornerstone of many ecosystem processes (Clarke et al. 2026) and accumulates in the absence of herbivory, serving as an important indirect pathway by which arthropod herbivores and soil conditions change. Ungulates directly impact thatch accumulation through consumption (Kjaer et al. 2024) and indirectly affect its quality by preferentially selecting palatable over unpalatable species (Ramirez et al. 2021; Rhodes et al. 2025), thereby altering the arthropod community structure that utilizes it. Detrital and soil faunal communities, in turn, support a wide range of ecosystem services through their role in decomposition and soil processes (Clarke et al. 2026). Litter alters microhabitats for aboveground and belowground fauna by providing habitat and food and by interacting abiotically with the soil surface (Clarke et al. 2026). Given that ungulates reduce litter accumulation (Kjaer et al. 2024; Ramirez et al. 2021; Rhodes et al. 2025) and change litter quality (Fornara and du Toit 2008), ungulates may indirectly alter arthropod communities in the litter (Ramirez et al. 2021). Moreover, removing leaf litter can increase soil susceptibility to compaction and raise soil temperature (Shah et al. 2017), with additional cascading effects on detrital communities.

Ungulate activity can compact soil, limiting root growth and altering water and nutrient absorption (Ramirez et al. 2021; Shah et al. 2017; Whalley et al. 1995). Physical perturbation reduces porosity and alters pore‐size distribution, reducing absorption of high‐intensity rainfall and, over time, leading to anaerobic conditions (Whalley et al. 1995). Despite adequate water and nutrients, plants may grow more slowly and develop shallower roots in compacted soils, suggesting that soil compaction limits root elongation rates (Shah et al. 2017; Whalley et al. 1995). Penetration resistance greater than 2 MPa (300 psi) can indicate severe soil structural damage, influencing effective bulk density, macropore volume, and water permeability (Beylich et al. 2010). In sum, soil compaction affects the proportions of water and air, limiting plant growth (Beylich et al. 2010).

Our goal was to assess the direct and indirect impacts of ungulates on vegetation, litter, and associated arthropods, pathogens, and detritivore communities over 8 years of ungulate exclusion. Because large herbivores can also restore ecosystem functions when reintroduced, we complemented long‐term ungulate exclusion with subsequent fence removal to test early rewilding effects. The study design included three treatments: (a) ungulates always present (unfenced controls), (b) ungulates always excluded (fenced), and (c) ungulates reintroduced, hereafter “rewilded” (fencing removed). We hypothesized several key ecological shifts arising from these treatments. When ungulates were excluded, we predicted (1) changes in the woody and herbaceous plant community toward palatable communities, (2) an increase in plant biomass, litter abundance, and lower soil compaction, (3) simplification of litter arthropod communities, and (4) increased herbivorous arthropod and grass pathogens. Conversely, we predicted that when ungulates were rewilded, all of these factors would track toward the unfenced controls. We also predicted that all buffelgrass volume would increase across the precipitation gradient. Finally, we predicted that precipitation and treatment would interact with each response measured.

## 2 Materials and Methods

### 2.1 Study Site

The study was conducted at the Mpala Research Centre (MRC), a private conservancy in Laikipia County, central Kenya (0.292°N, 36.896°E). MRC is a research facility and working ranch situated along the Ewaso Ng'iro River in a semi‐arid tropical savanna in the rain shadow of Mt. Kenya. The MRC covers approximately 20,000 ha and is surrounded by local community lands, large ranches, and conservancies. The savanna vegetation is characterized by dominant trees and shrubs, including *Acacia mellifera (*= *Senegalia mellifera*), *A. brevispica (*= *Senegalia brevispica*), *A. etbaica (*= *Vachellia etbaica), Croton* spp., and diverse grasses, e.g., *Cenchrus ciliaris, Aristida kenyensis, Cynodon plectostachyus, Eragrostis tenuifolia*, and *Heteropogon contortus* . The long axis of MRC (30 km) stretches across a rainfall gradient with a 50% increase from lower (400 mm) to higher (600 mm) precipitation (Figure 1). The rainy season at MRC is bimodal, with the “long‐rains” season being the larger seasonal peak of primary productivity periods associated with Kenya's rainy seasons, generally in March to May. Historical weather data is available through the MRC (Caylor et al. 2026). Six sites along this precipitation gradient were selected, with two sites in each of the northern, central, and southern regions of the MRC. We classified the six sites into xeric, mesic, and hydric categories based on their long‐term precipitation regimes along the north‐to‐south aridity gradient. We conducted our research at sites with red sand soils (15% clay and 74% sand) and deep transition soils, ranging from well‐drained sandy clay loam to clay loam (Ahn and Geiger 1987).

**FIGURE 1:**
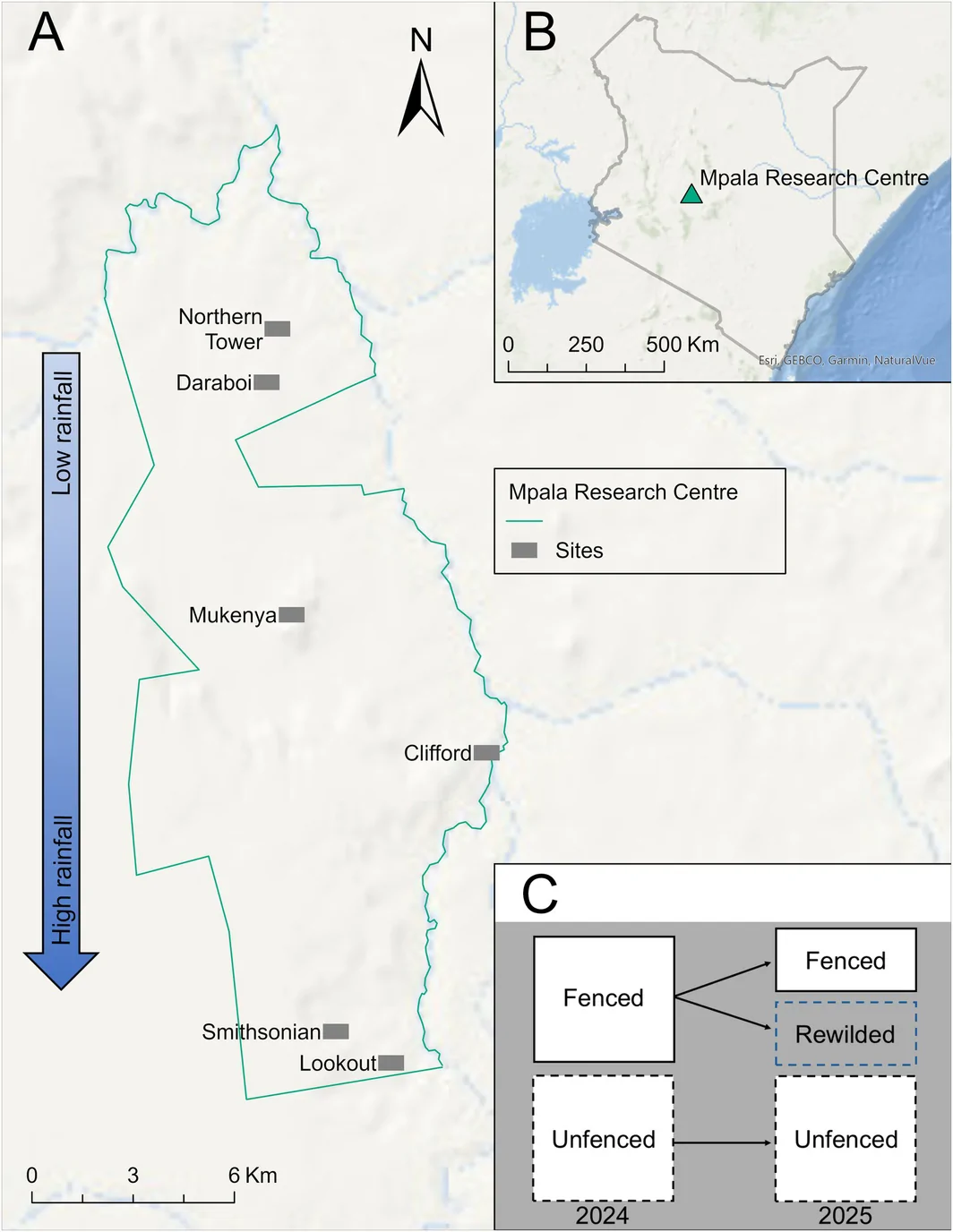
Map and conceptual design of original experiment. (A) Mpala Research Centre and study sites. (B) Inset map of Kenya. (C) Fencing treatments: Fenced for 8 years, rewilded 9 months prior, and unfenced.

In May 2017, we delineated two 50 × 50 m paired plots at each site and erected 2.5 m tall electrified fencing to exclude ungulates in one plot, paired with a fenceless plot that allowed normal access. In September 2024, half of the 50 × 50 m fence was removed to allow rewilding after 7 years of exclusion. Sites were surveyed in June 2024, before fence removal (12 sites), and again in June 2025, 9 months after rewilding (18 sites).

### 2.2 Ungulate Communities

MRC had approximately 3000 livestock grazing on 20,000 ha at low to moderate stocking intensities, comprising a mix of Zebu/Boran cattle ( *Bos taurus indicus* ), camels ( *Camelus dromedarius* ), goats ( *Capra hircus* ), and sheep ( *Ovis aries* ). Livestock grazing followed traditional pastoralism, centered on bomas (overnight holding pens) that are moved after several months but often reused every few years, leaving a distinctive patch of fertile soil and a radiating gradient of grazing pressure (Augustine 2004). Mpala Research Centre regularly records the presence of 18 ungulate species (herein, terrestrial artiodactyls, perissodactyls, and proboscideans): *Bos taurus* Linnaeus, 1758, *Equus quagga* Boddaert 1785, *Aepyceros melampus* Lichtenstein 1812, *Loxodonta africana* Blumenbach 1797, *Giraffa reticulata* De Winton 1899, *Equus quagga burchellii* Gray 1824, *Syncerus caffer* Sparrman 1779, *Equus grevyi* Oustalet 1882, *Ovis aries* Linnaeus 1758, *Madoqua guentheri* Thomas 1894, *Kobus ellipsiprymnus* Ogilby 1833, *Tragelaphus strepsiceros* Pallas 1766, *Taurotragus oryx* Pallas 1766, *Phacochoerus africanus* Gmelin 1788, *Capra hircus* Linnaeus 1758, *Nanger granti* Brooke 1872, *Raphicerus campestris* Thunberg 1811, and *Hippopotamus amphibius* Linnaeus, 1758 (Goheen et al. 2013; Rhodes et al. 2022; Riginos et al. 2012; Young et al. 1998).

### 2.3 Plant Communities

#### 2.3.1 Woody Community

To assess ungulate impacts on woody vegetation, we assessed woody vegetation inside and outside each fence in 2024 and again in 2025, including the rewilding treatment. We identified each individual to species level that was at least 1 cm in diameter at 1.3 m in height. We then tagged them and measured their diameter at 1.3 m height, as well as the width and length of their canopies. The foliar area was calculated by combining the length (L) and width (W) into the area of an ellipse (πLW/4) and was then used to assess their community composition in ordination.

#### 2.3.2 Herbaceous Community

Within each plot, six 1 × 1 m subplots were set up both in fenced and unfenced plots, for a total of 72 subplots. In the rewilded treatment of 2025, half of the plots from the fenced plot of 2024 were opened up for access and treated as rewilded. The subplot corners were marked with two 1 cm diameter, 0.5 m tall iron reinforcing bars set into the ground at opposite corners of the plot, and a numbered aluminum tag was attached to one of the corners. We recorded the identities of all herbaceous species and their foliar cover to the nearest 0.1%. Additional cover types were litter and bare ground.

#### 2.3.3 Buffelgrass Productivity

To evaluate buffelgrass productivity, we measured buffelgrass basal area and height. We chose the two largest buffelgrass tussocks for each subplot, recorded the basal circumference to the nearest 1 cm, and calculated the area of a cylinder in L using the height and circumference $V=\frac{C^{2}h}{4\pi }$. The height of the tallest leaf was recorded in centimeters. We averaged these metrics across the two largest buffelgrass tufts to represent the buffelgrass productivity in the subplot. The subplots were sampled during June, after the long rains in 2024 and 2025.

#### 2.3.4 Arthropods and Pathogens on Buffelgrass Leaves

We surveyed leaf damage as a percentage and categorized it by arthropod and pathogen damage classes. Within each fencing treatment, we randomly selected 10 tussocks for assessment from the nearest neighbor to the subplots, excluding those within the subplots. From each buffelgrass tussock, we randomly selected 10 mature leaves and estimated leaf damage as a percentage of the leaf that had damage or was missing due to external chewing, but not grazing. We then assigned leaf damage to a damage class indicating the manner of damage inflicted on the plants: external chewers, internal chewers, or pathogens. External chewers were arthropods with chewing mouthparts that remove large discrete sections of leaf tissue, often Orthoptera. Internal chewers were defined as causing damage visually consistent with leaf‐mining, or piercing/sucking insects' feeding habits that chew internal tissues in visible tracks or evacuated spots. Pathogen damage included foliar symptoms visually classified as leaf spots, rusts, or powdery mildew seen on the sampled leaf material.

### 2.4 Litter and Soil Invertebrates

#### 2.4.1 Litter Arthropods

Litter was surveyed as percent cover and collected from a 1 m^2^ quadrat centered on a buffelgrass plant, selected at random from inside and outside the exclosures. We collected litter until we obtained 500 g of fresh material from each of the 12 sites (2024) and 18 sites (2025). The litter was placed in a paper bag and weighed in the field to relate percent cover to fresh mass. Litter samples were placed in a Winkler‐Moczarski extractor—a coarse‐mesh muslin bag, suspended inside a large, closed muslin bag over a 100‐ml collection bottle containing 50 mL of 80% ethanol (Besuchet et al. 1987). Daily, the litter material was gently mixed by hand to ensure the fauna remained active, and dropping into the collection jar was encouraged (Besuchet et al. 1987; Parr and Chown 2001). Arthropod abundances were standardized by collection area, with the number of arthropods per m^2^ analyzed.

#### 2.4.2 Arthropod Identification to Family and Functional Group

For arthropods from the fenced and unfenced plots in 2024, we used an iterative workflow to determine arthropod identities and functional feeding groups. With a team of two to three trained parataxonomists, we sorted arthropods from litter samples into morphospecies differentiated by consistent morphological characteristics. We identified them to the lowest possible taxonomic level, recognizing that most taxa we encountered are undescribed (Morrison et al. 2023). We counted and imaged all specimens under a Leica Wild M8 dissecting microscope (Germany). We further validated arthropod specimens using rearing data focused on green buffelgrass vegetation (Morrison et al. 2023), consultation with taxonomic experts, and life history literature. With this approach, we inferred the functional feeding group of arthropod species or morphospecies (herbivore, predator, parasitoid, or detritivore). We deposited all specimens in 100% ethanol vials at the University of Texas Insect Collection (UTIC); morphospecies images are available in the supplement.

#### 2.4.3 Soil Abiotic Characteristics

We measured soil depth at high compaction (> 2.0 MPa) and litter depth in 2025 within the fenced, rewilding, and unfenced plots across all sites. Soil depth was measured using a FieldScout SC900 Soil Compaction Meter (Spectrum Technologies Inc., Aurora, IL, USA). We measured the depth of soil until compaction reached 2 MPa (300 psi). This depth was used as an index of soil depth relevant to root elongation. We measured litter depth by inserting a ruler vertically through the litter layer until it touched the mineral soil layer. Across all 6 sites, we took 12 soil depth measurements and 12 litter depth measurements within each experimental plot (fenced, rewilding, and unfenced).

### 2.5 Statistical Analysis

We addressed six related questions using two analytical frameworks: canonical correspondence analysis (CCA) to assess impacts of ungulate herbivory and precipitation on plant community composition, and linear mixed‐effects models to test the effects of fencing, precipitation regime, and their interaction on buffelgrass volume and natural enemy communities. Analyses were conducted in Program R 4.5.2 (R Core Team 2025).

### 2.6 Plant Community CCA Ordinations

To understand the influence of ungulate herbivory and precipitation on plant community composition, we used canonical correspondence analysis (CCA), with fencing and precipitation regime as categorical predictors for woody and herbaceous plant community responses. We conducted four CCAs in total, separating them by woody and herbaceous plant community types and by pre‐ and post‐rewilding survey periods (2024 and 2025, respectively). Fencing in 2024 had two levels in CCAs (fenced and unfenced), with a rewilding category added for 2025 rewilding CCAs. Foliar canopy area was used as the response metric in woody plant community CCAs, and foliar cover was used as the response metric in herbaceous plant community CCAs.

To reduce the influence of extremely rare taxa, we excluded species that occurred in < 2% of plots throughout the study area within each sampling period before conducting the CCAs (McCune et al. 2002) (Table S3). This resulted in 9 woody species in each of the pre‐ and post‐rewilding analyses, and 62 and 44 herbaceous species in the pre‐ and post‐rewilding analyses, respectively. Woody plant community canopy data were right‐skewed due to the dominance of larger canopy species. We applied Hellinger transformations on canopy area before conducting both ordinations to reduce the influence of the dominant species while preserving absent values and the relative contributions of each species on community composition. We also compared these models to untransformed and log_(x+1)_ transformed models to ensure robustness and consistency of treatment effects on plant community structure in the ordination space. CCA results were qualitatively consistent across transformations.

CCAs were computed with chi‐square distances, with total inertia partitioned into constrained and unconstrained components. We identified two dominant constrained CCA axes to display the relationships between the respective plant communities, fencing, and precipitation regimes. We assessed the statistical significance of the canonical axes using permutation tests (*n* = 999) under a reduced model, with stepwise forward testing of axes. We also explored ungulate‐exclusion treatment by precipitation regime interactions. All analyses for the CCAs were conducted using the “vegan” package (Oksanen et al. 2023).

### 2.7 Linear Mixed‐Effect Models to Assess the Impact of Fencing and Precipitation on Buffelgrass Traits, Foliar Arthropods, Foliar Pathogens, Litter, and Soil Biota

For response variables evaluated with linear mixed‐effects models, we modeled fencing treatment, precipitation regime, and their interaction as fixed effects. All linear mixed‐effects models were fit by restricted maximum likelihood (REML) using the “nlme” package (Pinheiro et al. 2021). We analyzed 2024 and 2025 separately: in 2024, fencing was either fenced or unfenced; in 2025, there were three fencing treatments: fenced, rewilded, and unfenced. We assessed normality and outliers visually using histograms of model residuals and Cleveland boxplots, respectively (Zuur et al. 2010). For each response variable, we addressed heterogeneity of variance using a variance identity structure to allow variance within each sampling unit.

### 2.8 Buffelgrass Vegetation Volume

We used linear mixed‐effects models to assess the effects of fencing, precipitation regime, and their interaction on buffelgrass volume. The identity structure allowed variance within each plot. Plot was included as a random variable.

### 2.9 Arthropods and Pathogens on Buffelgrass Leaf Tissue

We used linear mixed‐effects models to assess the impact of fencing, precipitation regime, and their interaction on total damage, pathogen damage, external chewer damage, and average damage per leaf. The identity structure allowed variance around each sampled tussock. Tussock was included as a random variable.

### 2.10 Litter Arthropods

We used linear mixed‐effects models to assess the impact of fencing, precipitation regime, and their interaction on the total number of arthropods per plot. The identity structure allowed variance around each tussock. Tussock was included as a random variable. We used counts of arthropods identified to morphospecies at the family level to examine diversity and abundance, and analyzed morphospecies' functional groups to infer tritrophic dynamics. We could not use June 2025 fenced, unfenced, and rewilded data because of the timing of permitting.

We examined the effects of fence treatment, precipitation regime, their interaction, and the continuous covariate litter depth on arthropod abundance using linear mixed‐effects models. We included site as a random intercept to account for spatial autocorrelation within the study. The random intercept variance was negligible, indicating minimal site‐level variation. We specified heterogeneous residual variances across precipitation regimes using a variance function to account for unequal error variances and improve model fit.

### 2.11 Soil Depth and Compaction

We examined the effects of the fence treatment, precipitation, and their interaction on soil depth and compaction using linear mixed‐effects models. We nested the fence within the site as a random effect. To account for heterogeneity of variance across precipitation regimes, we used an identity structure.

## 3 Results

### 3.1 Buffelgrass Morphology 2024

Buffelgrass vegetative volume in unfenced areas in 2024 was reduced by approximately 5.5 L per m^2^ compared to fenced areas (*F*_1, 55_ = 7.58, *p* = 0.008). Precipitation significantly impacted buffelgrass volume (*F*_2,3_ = 5.6, *p* = 0.01), with mesic areas supporting lower volumes than xeric sites. The interaction between fence and precipitation was nonsignificant (*F*_2,55_ = 0.84, *p* = 0.44), indicating that fence effects on grass volume are consistent across all precipitation zones.

### 3.2 Buffelgrass Morphology 2025

In 2025, fence treatment significantly affected buffelgrass volume (*F*_2,259_ = 7.6, *p* < 0.001), with both rewilded and unfenced plots showing reduced volumes compared to the fenced treatment (−7.6 ± 1.4 SE and −7.4 L ±1.2 SE, *p* < 0.001, respectively). Precipitation regime main effect was not significant. The fence by precipitation interaction was highly significant (*F*_4,259_ = 8.5, *p* < 0.001), driven by fencing in the hydric precipitation regime having very high volumes (Figure 2).

**FIGURE 2:**
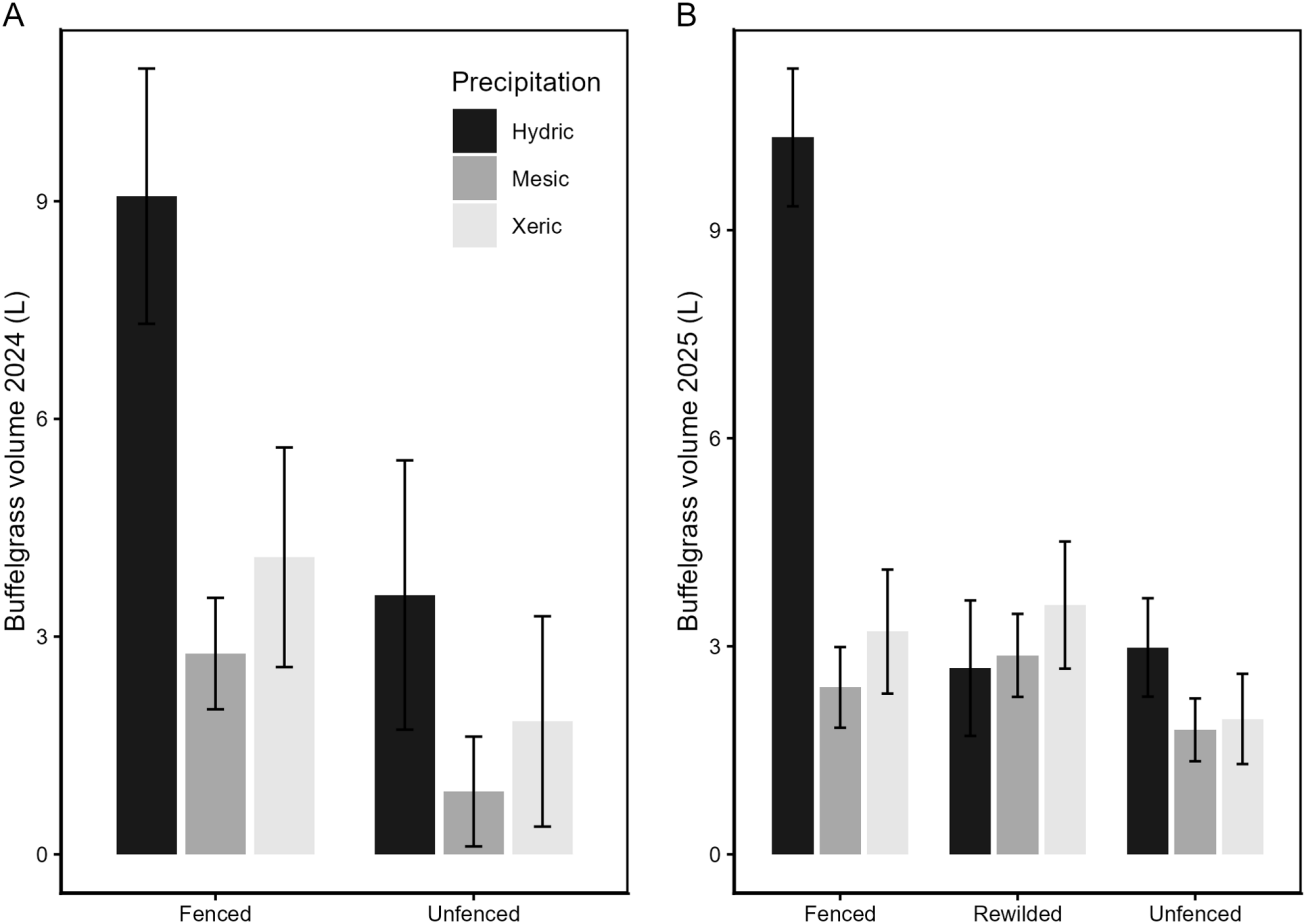
Buffelgrass vegetation volume (L m^−2^) is shown for 2024 and 2025 in panels (A, B), respectively. The means and standard errors are model estimates. Error bars represent ±1 standard error.

### 3.3 Plant Communities

#### 3.3.1 Woody Plant Community After Rewilding 2025

The model included canopy cover data for 9 woody plant species associated with fencing and precipitation regime (Table S1), resulting in 4 constrained axes. The total inertia of the species data was 8.0, with 0.77 (10%) explained by the constrained component, and 7.23 (90%) remaining unconstrained. The first three canonical axes were statistically significant (CCA1: *F* = 68.6, *p* = 0.001; CCA2: *F* = 29.6, *p* = 0.001; CCA3: *F* = 19.0, *p* = 0.001), with the first two axes accounting for 83.3% of the variation. A significant interaction between fencing and precipitation (*F* = 5.68, *p* = 0.001) indicated that the effect of fencing on community composition varied across precipitation regimes. Precipitation regime explained the largest portion of constrained variation (*F* = 46.4, *p* = 0.001), followed by fencing (*F* = 13.6, *p* = 0.001).

Species clearly clustered by treatment category along the primary canonical axes (Figure 3), indicating that both fencing and precipitation regime contributed to differences in woody species assemblages in 2025. This pattern was mainly driven by *Acacia gerardii* and *Euclea divinorum* having higher canopy cover in hydric sites, regardless of fencing treatment, and *Euphorbia kibwezensis* having higher canopy cover in fenced or rewilded xeric sites. *Acacia brevispica* also had higher canopy cover in fenced or rewilded sites, but in both xeric and mesic sites. Woody plant community data from fenced and unfenced treatments in 2024 are available in the supplement.

**FIGURE 3:**
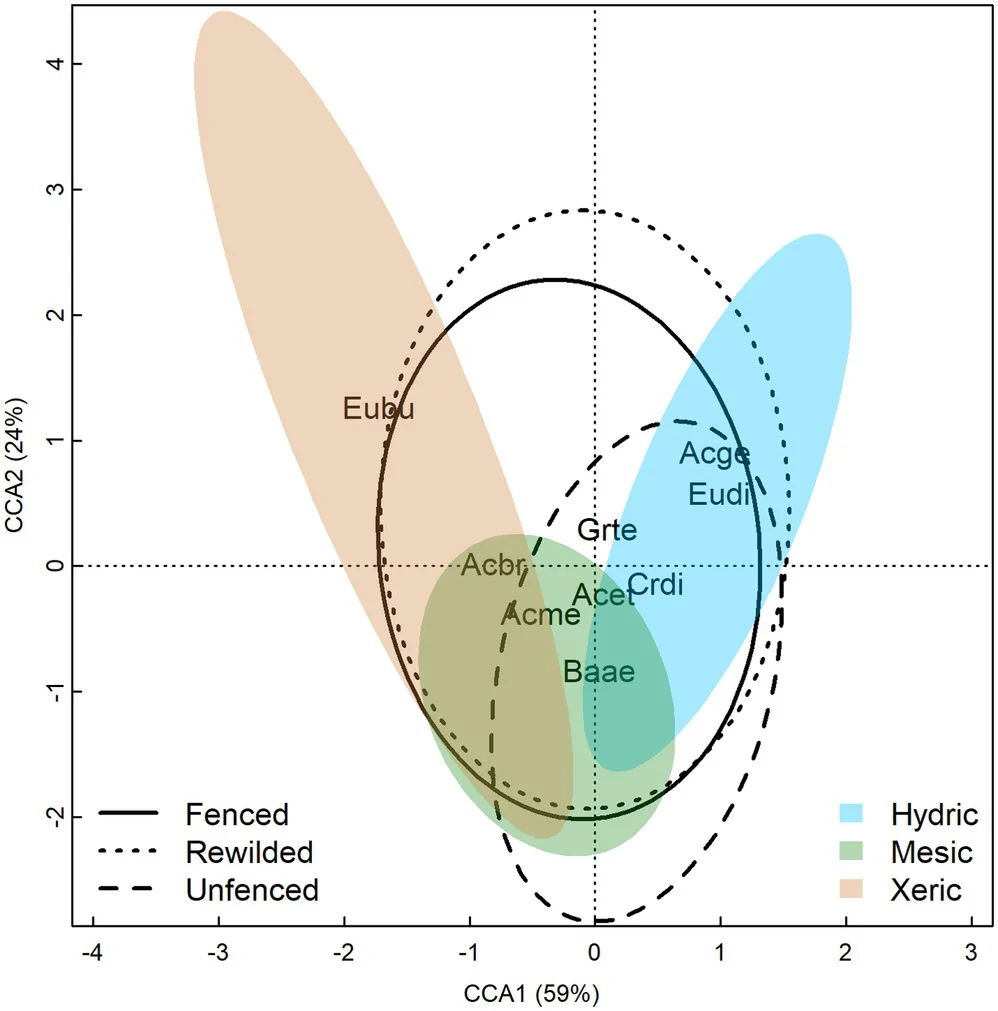
CCA denoting the woody plant species in 2025. Ellipses demonstrate groupings by fencing (dotted vs. solid lines) and precipitation regime in color.

#### 3.3.2 Herbaceous Plant Community After Rewilding 2025

The model included cover data of 44 herbaceous plant species associated with fencing and precipitation regime (Table S2), resulting in four constrained axes. The total inertia of the species data was 6.57, with 0.77 (12%) explained by the constrained component, and 5.8 (88%) remaining unconstrained. The first two canonical axes were statistically significant (CCA1: *F =* 3.99, *p* = 0.001; CCA2: *F =* 2.42, *p* = 0.016) and accounted for 79.3% of the variation. Subsequent axes did not add significant information (*p* > 0.4). Although the interaction between fencing and precipitation was insignificant (*F =* 1.07, *p* = 0.37), each predictor significantly contributed to species partitioning (*F* = 2.97, *p* = 0.001; *F* = 1.50, *p* = 0.009, respectively), as demonstrated along the primary canonical axes (Figure 4). Specifically, *Heteropogon contortus* and *Aristida kenyensis* had higher cover in hydric sites subjected to ungulate herbivory. *Bothriochloa radicans*, *Eragrostis tenuifolia* , *Eragrostis superba* , *Helichrysum glumaceum*, *Cyperus mariscus*, and *Aristida adoensis* had higher cover in hydric sites, regardless of fencing. Similarly, many species had higher cover in xeric sites, regardless of fencing, including *Evolvulus alsinoides* , *Gutenbergia cordifolia*, *Eragrostis rigidior*, *Euphorbia scarlatina* , *Asparagus africanus* , *Cyathula uncinulata*, *Barleria acanthoides*, and *Sporobolus festivus*. A few species had higher cover in fenced, mesic sites, including *Plectranthus caninus*, *Conyza stricta*, *Ruellia prostrata* , *Hibiscus micranthus*, *Chloris roxburghiana*, *Barleria trispinosa*, and *Cymbopogon pospischilii*. Regardless of precipitation regime, *Sehima nervosum* , *Sida ovata*, and *Crotalaria pycnostachya* were more common in unfenced and rewilded plots. *Cenchrus ciliaris* , *Justicia matammensis*, *Pennisetum stramineum*, *Ocimum gratissimum* , and *Melhania ovata* were the least influenced by the constrained predictor gradients, unaffected by fencing and precipitation regime. Herbaceous plant community data from the 2024 fenced and unfenced treatments are available in the supplement.

**FIGURE 4:**
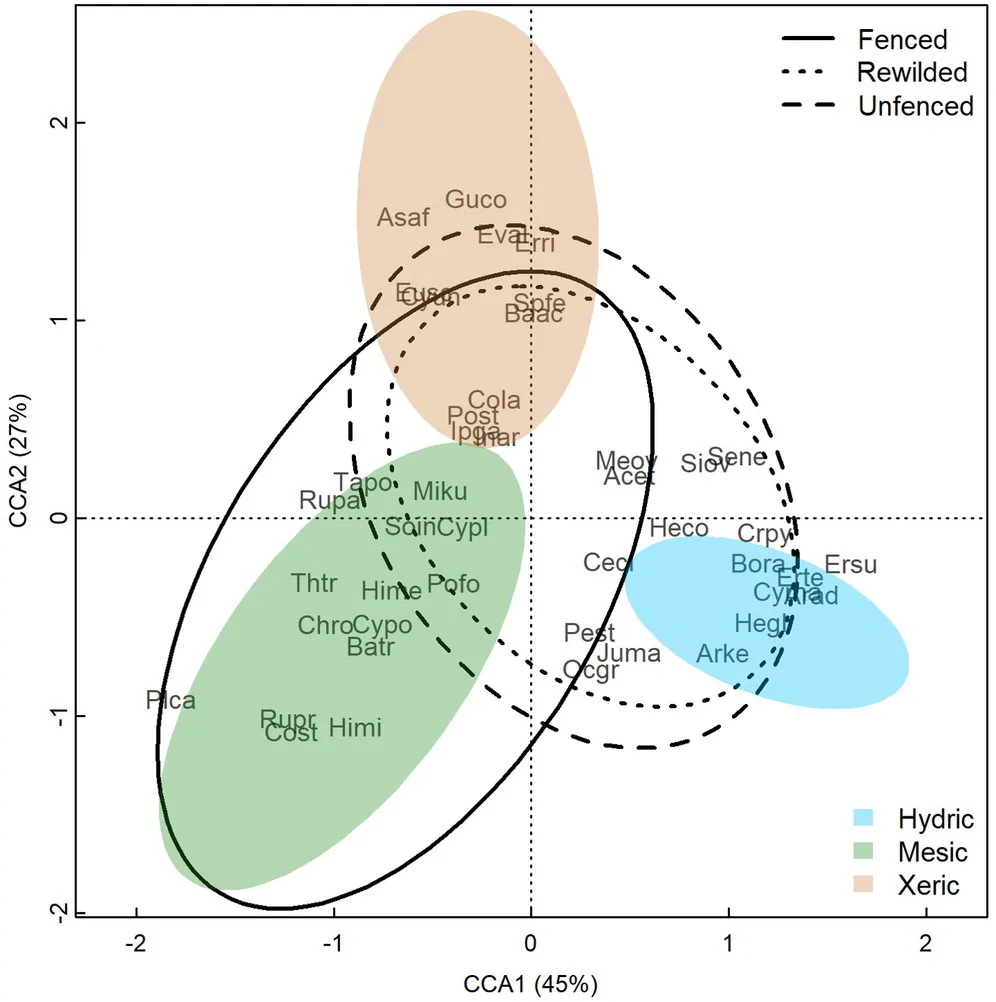
CCA denoting the herbaceous plant species in 2025. Ellipses demonstrate groupings by fencing (dotted vs. solid lines) and precipitation regime in color. Species labels were jittered to improve readability while preserving relative positions in ordination space.

### 3.4 Leaf Damage

#### 3.4.1 Total Leaf Damage 2024

Fencing significantly increased total leaf damage (*F*_1,105_ = 28.2, *p* < 0.001), with fenced plots experiencing nearly 3 times more total damage (mean = 23% ± 2.2% SE) compared to unfenced plots (mean = 8.8% ± 3.1% SE). Precipitation regime alone did not significantly influence total damage (*F*_2,105_ = 0.7, *p* = 0.5). However, a significant interaction between precipitation and fencing emerged (*F*_2,105_ = 3.1, *p* = 0.05), given that fenced and unfenced plots were more similar under mesic conditions (Figure 5A).

**FIGURE 5:**
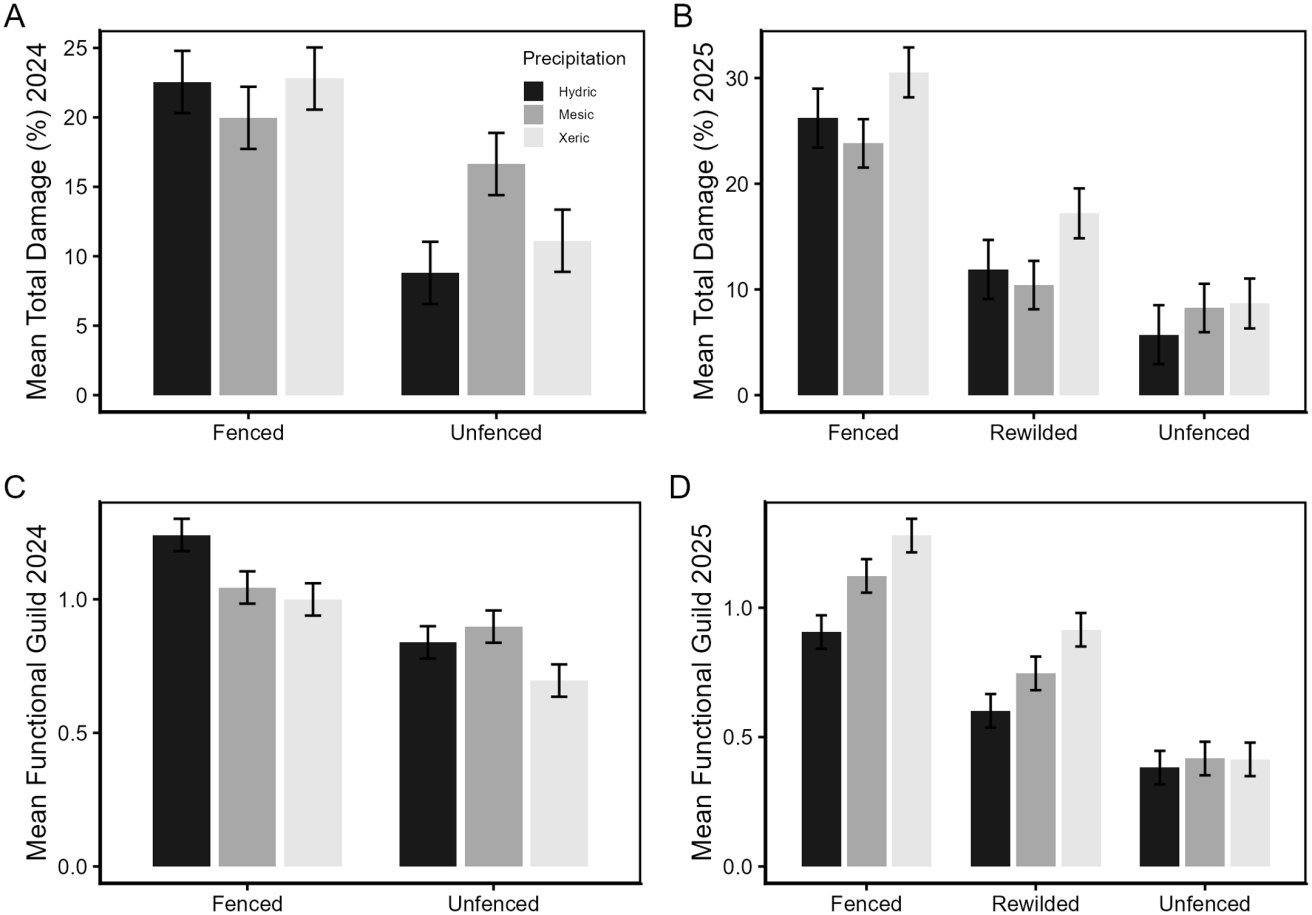
Mean total damage in 2024 and 2025, and mean functional guild in 2024 and 2025 are shown in panels (A–D), respectively. Error bars represent ±1 standard error.

#### 3.4.2 Pathogen Damage 2024

Fencing strongly increased pathogen damage (*F*_1,105_ = 17, *p* < 0.001), with fenced plots averaging 20.6% ± 2.2% SE compared to unfenced plots at 7.9% ± 3.15% SE, a nearly 3‐fold increase. The precipitation regime was nonsignificant (*F*_2,105_ = 0.85, *p* = 0.43). The precipitation by fencing interaction approached significance (*F*_2,105_ = 2.9, *p* = 0.06), with mesic unfenced sites showing substantially elevated pathogen damage relative to hydric unfenced sites (10.8%; *t* = 2.41, *p* = 0.02).

#### 3.4.3 External Chewer Damage 2024

External chewer damage patterns differed notably from pathogen damage because they were driven more by precipitation regimes. Precipitation regimes significantly affected external chewer damage (*F*_2,105_ = 5.2, *p* = 0.007), with xeric sites showing elevated damage with 3.2% higher absolute leaf damage due to external chewers than in hydric sites (*t* = 3.2, *p* = 0.002). Fencing had no significant main effect on external chewer damage (*F*_1,105_ = 2.2, *p* = 0.15), and the precipitation regime by fencing interaction was nonsignificant (*F*_2,105_ = 1.2, *p* = 0.3).

#### 3.4.4 Damage Class Count 2024

Fencing significantly increased richness of damage class in 2024 (*F*_1,105_ = 33, *p* < 0.001), with fenced plots supporting 1.24 ± 0.06 SE damage classes per leaf compared to 0.84 ± 0.09 SE in unfenced plots (Figure 5C). Precipitation also significantly affected damage class counts (*F*_2,105_ = 5.2, *p* = 0.007), with both mesic (*β* = −0.20, *t* = −2.3, *p* = 0.02) and xeric (*β* = −0.24, *t* = −2.8, *p* = 0.006) sites showing reduced damage class counts per leaf relative to hydric precipitation regime conditions. A precipitation by fencing interaction trend emerged (*F*_2,105_ = 2.3, *p* = 0.11), driven by damage class increase in mesic unfenced sites (interaction *β* = 0.26, *t* = 2.11, *p* = 0.04).

#### 3.4.5 Leaf Damage After Rewilding 2025

Fencing significantly increased total leaf damage on buffelgrass in 2025 (*F*_2,162_ = 48, *p* < 0.001), while precipitation regime also showed a significant main effect (*F*_2,162_ = 3.5, *p* = 0.03). However, the interaction between precipitation and fencing was not significant (*F*_4,162_ = 0.66, *p* = 0.62), indicating that fencing effects on damage were consistent across precipitation regimes. Fenced areas exhibited substantially higher leaf damage (26% ± 2.8% SE leaf area per leaf) compared to both recent rewilding (11.9% ± 3.9% SE leaf area, *t* = −3.6, *p* < 0.001) and long‐term unfenced areas (5.7% ± 3.9% SE leaf area, *t* = −5.2, *p* < 0.001) (Figure 5B).

#### 3.4.6 Pathogen Damage After Rewilding 2025

Pathogen damage in buffelgrass leaf tissue was significantly affected by both precipitation regime and fencing (*F*_2,162_ = 11, *p* < 0.001 and *F*_2,162_ = 26, *p* < 0.001, respectively), with a significant interaction between these factors (*F*_4,162_ = 3.4, *p* = 0.01). Fenced sites in hydric zones showed the highest pathogen damage (20.6% ± 1.8% SE, leaf area per leaf), while mesic and xeric precipitation regimes reduced damage to 9.9% ± 2.9% SE and 6.6% ± 2.9% SE, a reduction of damage by a half and 2/3rds, respectively. Compared with fencing, recent rewilding and unfencing reduced leaf area damage to 6.4% ± 2.6% SE and 4.3% ± 2.6% SE, respectively, representing 69% and 79% reductions in area, respectively. However, the interaction revealed that ungulate‐mediated pathogen suppression was most pronounced in wet conditions. In the two hydric sites, unfenced sites experienced 80% lower damage than fenced sites (4.3% ± 3.6% SE vs. 21% ± 3.6% SE). In contrast, in xeric zones, the ungulate effect was substantially diminished (0.54% ± 3.6% SE vs. 6.7% ± 3.6% SE for fenced sites).

#### 3.4.7 External Chewers After Rewilding 2025

External chewer damage was significantly affected by both precipitation regime and fencing (*F*_2,162_ = 4.9, *p* = 0.008 and *F*_2,162_ = 11, *p* < 0.001, respectively), but without a significant interaction (*F*_4,162_ = 0.76, *p* = 0.55). Fenced sites in hydric zones showed baseline external chewer damage of 8.2% ± 2.9% SE leaf area damaged, with mesic and xeric precipitation regimes increasing leaf area damage to 14.1% ± 2.9% SE and 13.9% ± 2.9% SE (or by 73% and 71%, respectively). Rewilding and unfenced treatments reduced leaf area damage to 9.5% ± 2.9% SE and 3.0% ± 2.9% SE, respectively (33% and 79%, respectively), with these ungulate effects consistent across all precipitation regimes.

#### 3.4.8 Damage Classes Per Leaf After Rewilding 2025

The number of functional damage classes per leaf varied significantly with both precipitation regime and fencing (*F*_2,162_ = 10, *p* < 0.001; *F*_2,162_ = 87, *p* < 0.001, respectively), with a significant interaction effect (*F*_4,162_ = 2.0, *p* = 0.01). Fenced plots in xeric conditions supported the highest damage class counts with 1.28 ± 0.06 SE classes per leaf. In comparison, unfenced plots maintained a 72% lower damage class count, or 0.36 ± 0.09 SE damage class types per leaf (Figure 5D). Ungulate suppression of damage classes was not significant across precipitation regimes (*F*_4,162_ = 2.01, *p* = 0.10). The negative association between unfenced plots and damage class count was stronger in xeric than in hydric conditions (*p* = 0.009). Unfenced plots had an estimated 0.52 ± 0.13 SE fewer damage classes in fenced plots under hydric conditions, and 0.86 ± 0.13 SE fewer guilds in xeric conditions.

#### 3.4.9 Litter Biota 2025

The fence treatments significantly affected arthropod abundance (*F*_2,5_ = 17, *p* < 0.001), while the precipitation regime did not (*F*_2,3_ = 0.45, *p* = 0.67) (Figure 6). Rewilding treatment increased arthropod abundance by 2.00 ± 0.67 individuals per plot relative to fenced areas (*t* = 3.0, *p* = 0.02), yet fenced and unfenced plots were similar. Litter depth had a significant positive effect on arthropod abundance (*F*_1,5_ = 8.7, *p* = 0.02). Each centimeter increase in litter depth increased arthropod abundance by 2.37 ± 0.80 individuals (*t* = 2.95, *p* = 0.02). The fence by precipitation regime interaction was not statistically significant (*F*_4,5_ = 2.7, *p* = 0.16).

**FIGURE 6:**
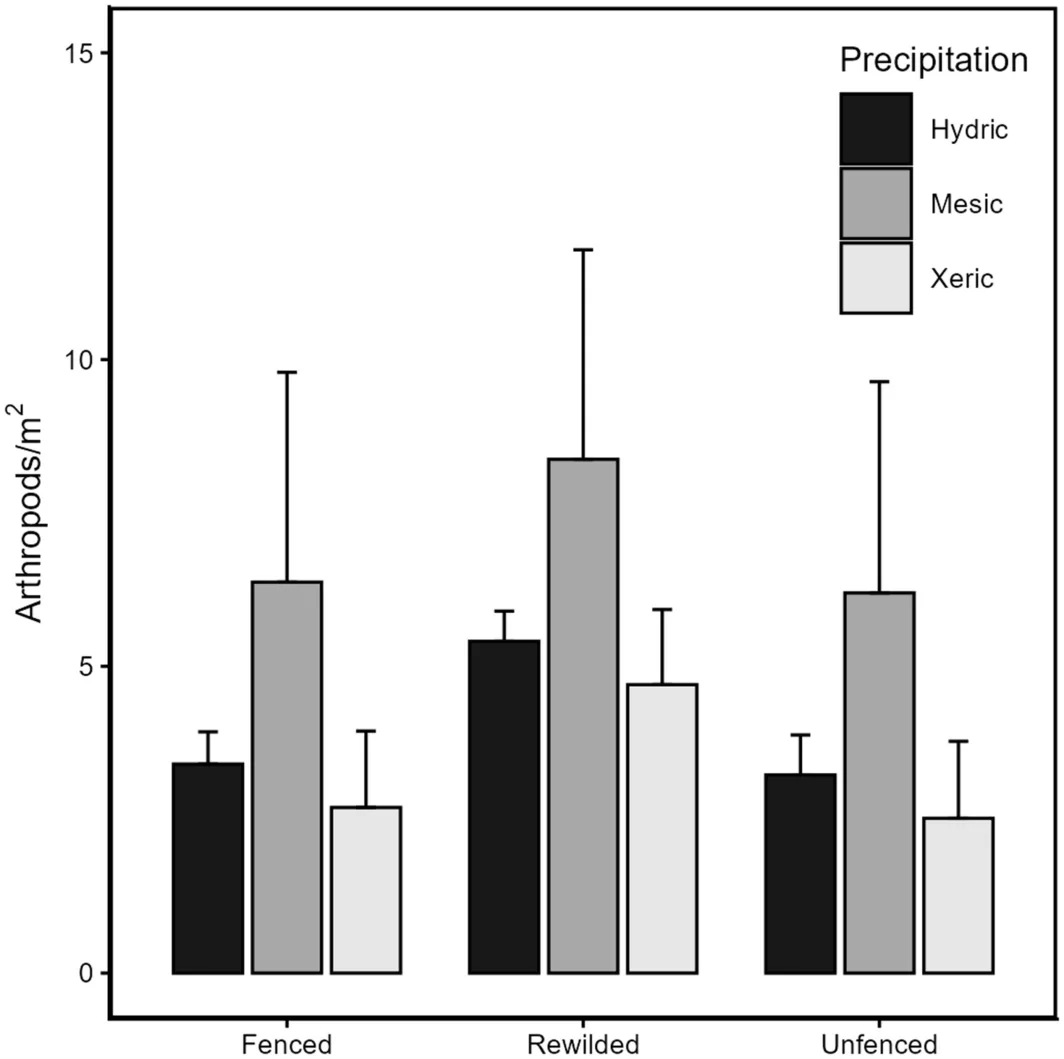
The number of arthropods per m^2^ is displayed across the fenced treatment groups, and the gray scale indicates the precipitation regime from June 2025 data. The error bar represents the standard error.

#### 3.4.10 Tri‐Trophic Arthropod Dynamics

Herbivore and detritivore abundances were stable across the fencing treatments and precipitation regimes; however, parasitoid abundances were negatively affected by the removal of ungulates, indicating a downgrading of trophic relationships in the absence of ungulates (Figure 7). Total arthropod abundance was not significantly affected by fencing (*F*_1,5_ = 0.02, *p* = 0.89), with similar counts observed between fenced and unfenced plots (*β* = 0.98, *t* = 0.14, *p* = 0.89). Precipitation regime also did not significantly influence total arthropod abundance. Similarly, fencing and precipitation did not significantly affect herbivore or detritivore abundance. Combined predator and parasitoid abundance showed no significant response to fencing (*F*_1,8_ = 0.30, *p* = 0.60) or precipitation (*F*_2,8_ = 1.50, *p* = 0.28). In contrast, parasitoid abundance alone was significantly affected by fencing (*F*_1,5_ = 15.87, *p* = 0.01), with unfenced plots supporting higher abundances than fenced plots with 10.75 ± 2.7 SE more parasitoids kg^−1^ (*p* = 0.01), while precipitation had no significant effect (*F*_2,3_ = 0.92, *p* = 0.49).

**FIGURE 7:**
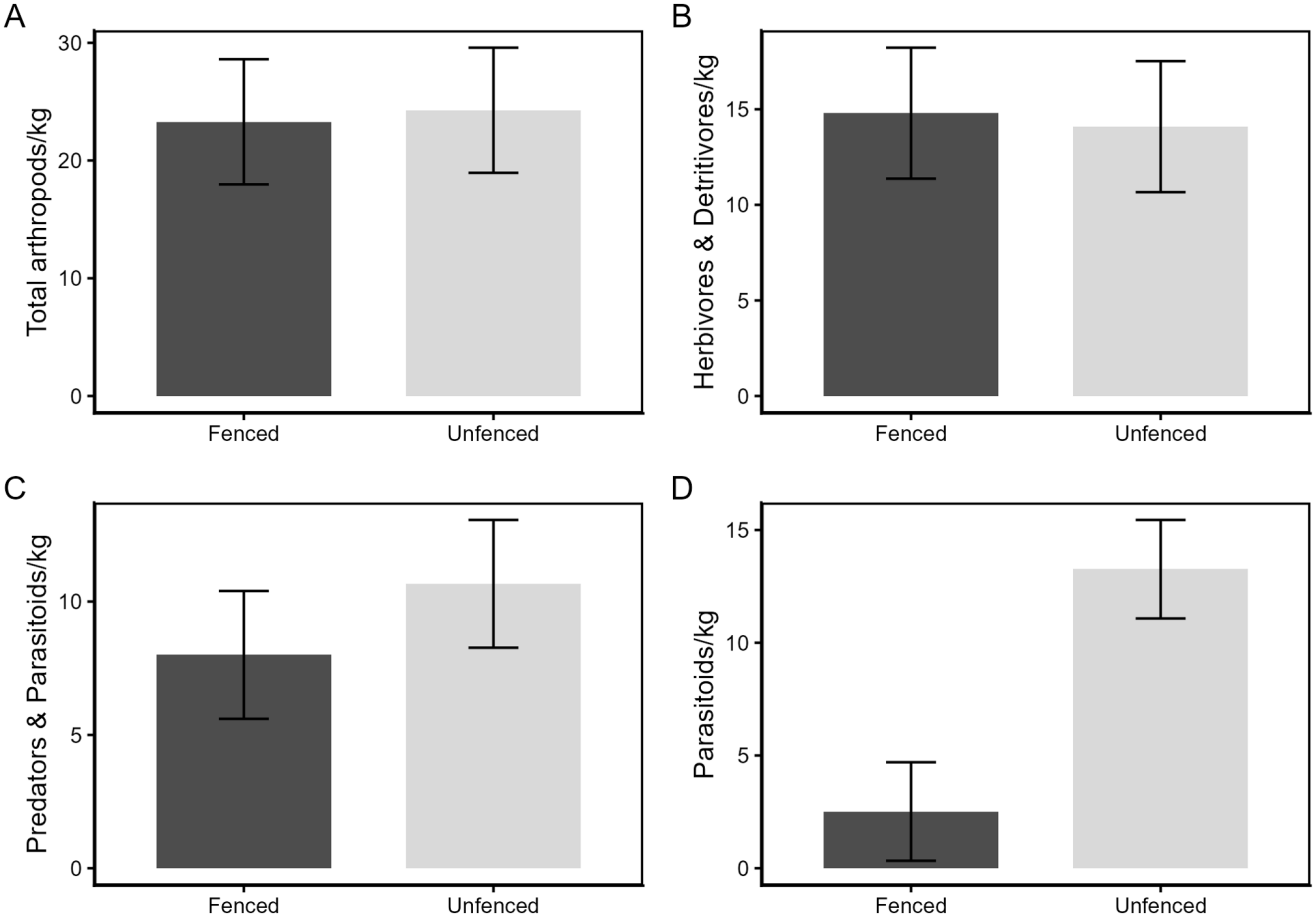
Winkler trap data are presented for the fenced and unfenced treatments in 2024. Arthropods are presented as the number per kg of litter, and the error bar is ±1 SE.

#### 3.4.11 Soil Abiotic

Soil depth until 2.0 MPa compaction varied significantly across fence treatments (*F*_2,6_ = 13, *p* = 0.007), with fenced areas showing greater soil depths of 11.3 cm ±3.4 SE on average compared to unfenced areas of 5.7 cm ±3.5 SE (Figure 8). However, the rewilded treatment showed minimal deviation from fenced conditions (0.69 cm difference). Precipitation regime did not significantly affect soil depth overall (*F*_2,3_ = 0.12, *p* = 0.89), nor did it interact significantly with fence treatment (*F*_4,6_ = 2.8, *p* = 0.13), suggesting that soil depth responses to rewilding were consistent across the three precipitation zones examined. Litter depth was significantly influenced by fence management (*F*_2,6_ = 31, *p* < 0.001), with unfenced areas showing 2.16 cm ±1.2 less litter accumulation compared to fenced areas. Precipitation regime had no significant effect on litter depth (*F*_2,3_ = 0.22, *p* = 0.82) and did not interact with fence treatment (*F*_4,6_ = 0.07, *p* = 0.99). Soil volumetric water content showed no significant response to fence treatments (*F*_2,6_ = 3.8, *p* = 0.08) or precipitation regime (*F*_2,3_ = 0.54, *p* = 0.63), with no significant interaction between these factors (*F*_4,6_ = 0.34, *p* = 0.85).

**FIGURE 8:**
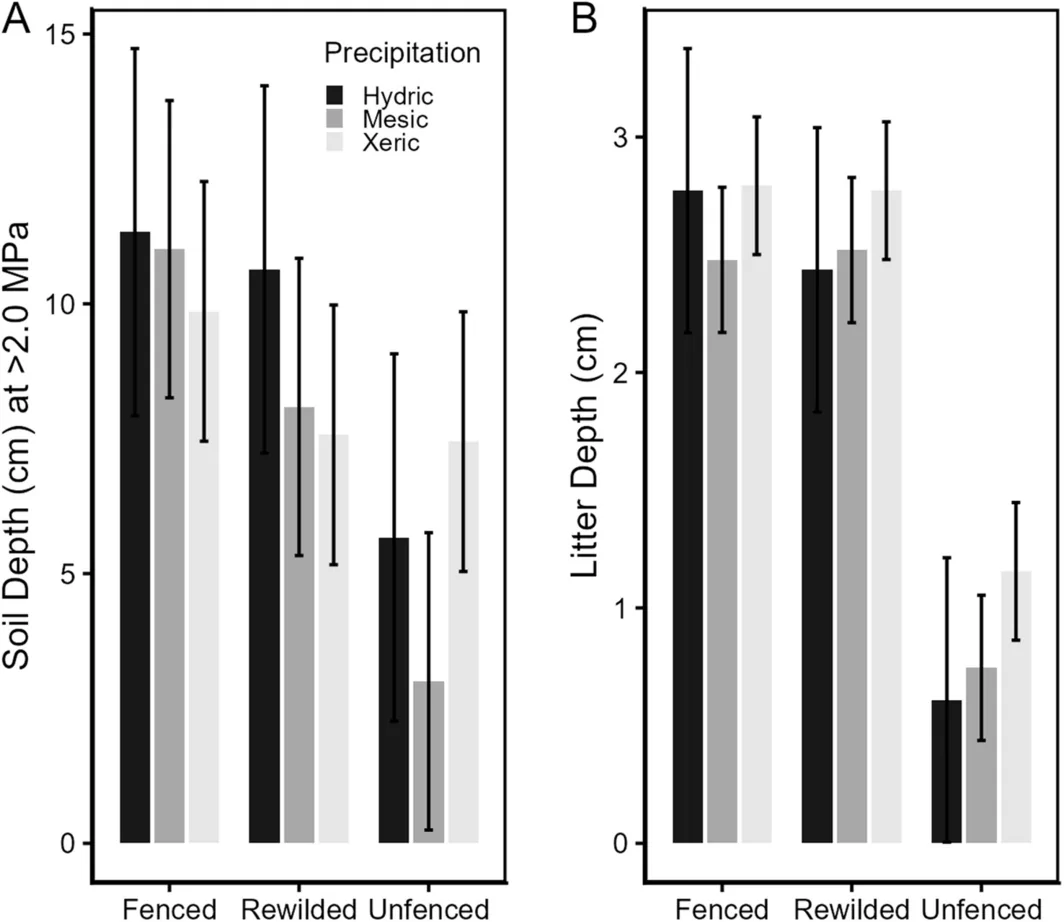
Soil depth at the depth where penetration resistance exceeded 2.0 MPa (≈300 psi), and litter depth is shown in panels (A, B), respectively. The means and standard errors are model estimates. Error bars represent ±1 standard error.

## 4 Discussion

Ungulates shape savanna vegetation and associated ecological communities, which change significantly in their absence (Coverdale et al. 2026; Pringle et al. 2023). Understanding how these changes develop and how they reverse is increasingly important as human activities continue to alter ungulate communities (Berger et al. 2020; Turner et al. 2022). Eight years of ungulate exclusion altered vegetation, foliar damage on buffelgrass, litter and litter‐associated arthropods, and soil conditions across a precipitation regime gradient. Removing fences then shifted some responses toward unfenced conditions within 9 months, although the magnitude and direction differed among vegetation and arthropod responses. These contrasts show both the breadth of ungulate effects and the different rates at which ecosystem components respond to rewilding.

Woody and herbaceous communities responded differently to rewilding. Woody species composition differed between fenced and unfenced plots after 8 years of exclusions, consistent with responses reported in other long‐term experiments (Goheen et al. 2018). Nine months after removal, however, the rewilded woody community had not shifted toward the unfenced community. The slower response likely reflects the timescales of woody plant growth, recruitment, and mortality in this savanna system (Coverdale et al. 2026). In contrast, the herbaceous community in rewilded plots shifted toward that of unfenced plots within 9 months, indicating a more rapid community response to trophic restoration.

Plant communities showed consistent but treatment‐specific patterns along precipitation and fencing gradients, highlighting the joint importance of abiotic filtering and ungulate herbivory interactions in shaping savanna vegetation (Schmitt et al. 2022). Across years, the precipitation regime explained the largest share of constrained variation, with fencing exerting an additional, context‐dependent effect, indicating that rewilding outcomes will depend strongly on how ungulate herbivory is filtered through underlying site resource availability, such as precipitation patterns (Goheen et al. 2013). Hydric precipitation regimes supported greater canopy cover of several woody dominants, including *Acacia gerardii* and *Euclea divinorum*, regardless of fencing, consistent with moisture‐driven increases in woody productivity and competitive ability over grasses. In contrast, xeric and mesic sites showed stronger fencing effects on canopy structure, suggesting that herbivore pressure interacts with resource limitation to structure woody assemblages through selective browsing and differential recruitment (Pansu et al. 2022).

Herbaceous assemblages showed similarly strong responses to precipitation regime and fencing, but with more pronounced species turnover and evidence of both grazer tolerant and grazer favored taxa. In hydric sites, many grasses (e.g., *Eragrostis superba* , *Aristida kenyensis*, and *Bothriochloa radicans*) were more common regardless of fencing, consistent with moisture‐driven increases in productivity and niche availability that can buffer communities against herbivory (Rhodes et al. 2024; Rhodes et al. 2023). In mesic and xeric sites, however, several species increased in fenced plots while others remained associated with ungulate presence, suggesting that species responded differently to fencing.

Together, these patterns imply that rewilding‐based savanna restoration should explicitly account for spatial heterogeneity in plant water availability, as restoration trajectories are likely to diverge across edaphic conditions and precipitation regimes. The rewilded plant communities indicate that herbaceous community responses to renewed herbivory may be relatively rapid but remain contingent on precipitation context and potentially species' functional traits. Increased cover of grazing or disturbance‐associated herbaceous species in unfenced plots, alongside the protection of browse‐sensitive woody species in fenced or rewilded sites, points to a mosaic of states maintained by resource gradients and herbivore pressure (Augustine et al. 2019).

The indirect effects of community interactions across taxa were highlighted by removing ungulates and observing the cascading impacts on how other taxa used buffelgrass. While insect herbivory in African savannas has received less attention (Maraia et al. 2024), our findings suggest that ungulate herbivory may regulate pathogen and insect damage, particularly in hydric habitats. When rewilded, the leaf damage fell within an intermediate range, suggesting some elastic response and resilience. Rewilding efforts that restore ungulate access provided substantial pathogen suppression, with the strongest benefits in wetter precipitation regimes where pathogenic pressure is naturally highest. While Emer et al. (2024) found that ungulate removal increases plant diversity and dilutes density‐dependent impacts of pathogens, we found that in a single palatable species, buffelgrass, ungulate absence led to higher pathogen and insect damage.

Our results suggest that ungulate effects on insects and pathogens of buffelgrass depend on precipitation. Changes in host‐plant abundance or structure may alter the resources available to insects and pathogens, and ungulates may filter for plant communities more palatable to arthropods (Fischer et al. 2022) and can interact with site water availability when ungulates have access (Torma et al. 2023). A shift to generalist arthropods in ungulate accessed sites can occur regardless of moisture availability (Torma et al. 2023), given that ungulates can directly perturb, consume (Gish et al. 2010; Gish et al. 2017), or alter the habitat of specialist insects (Rhodes et al. 2024). Water availability can further modify these relationships by changing plant growth and local conditions for insects and pathogens (Daskin and Pringle 2016; Torma et al. 2023). Reduced ungulate communities related to climate change and land use that, in turn, alter arthropods and pathogens are likely to be directly linked to reductions in ecosystem services in African ecosystems broadly (Archer et al. 2021).

Litter is increasingly recognized for its important role in ecosystem services (Clarke et al. 2026) and is directly and indirectly altered by ungulate activity through consumption, physical removal, or changes in litter chemistry (Fornara and du Toit 2008). Ungulates reduced litter depth, likely from mechanical disturbance and selective foraging on productive grasses, promoting thatch removal (Kjaer et al. 2024). We found that ungulate removal altered arthropod communities by reducing trophic complexity, as evidenced by declines in parasitoid abundance in leaf litter. Ungulates reduced litter percent and depth, which in turn altered arthropod numbers and community composition, showing the strong link ungulates can have on soil biota (Ramirez et al. 2021).

As megafauna continue to decline due to human activities and an increasing number of species face the uncertainty of extinction risk (Pringle et al. 2023), a deeper understanding of their direct and indirect relationships with plants, animals, and soil is needed. We showed that removing ungulates shifted communities at the leaf and litter levels, but that rewilding offered promise of the ecosystem's elasticity in returning to wild characteristics. Some aspects interacted with precipitation regimes, whereas others did not, suggesting varying effects of ungulate extinction and reduction across macro‐ and microclimatic conditions. Faunal communities in the deeper soils at the root layer of buffelgrass appeared more resilient to 8 years of exclusion and rewilding, suggesting that portions of the ecosystem are buffered at these shorter timescales. In conclusion, the loss of ungulates can shift natural enemy communities, increasing disease and pest pressure on important grasses, suggesting that a depauperate landscape could have indirect negative effects on agriculture and natural ecosystems alike, yet rewilding of ungulates allows the recovery of natural ecosystems.

## Author Contributions

**Aaron C. Rhodes:** conceptualization (equal), data curation (lead), formal analysis (lead), investigation (lead), methodology (lead), project administration (supporting), supervision (supporting), visualization (lead), writing – original draft (lead), writing – review and editing (lead). **Aimee Gaitho:** data curation (equal), investigation (supporting), methodology (supporting), supervision (supporting). **Kathryn E. Tisshaw:** data curation (equal), formal analysis (equal), validation (supporting), visualization (supporting), writing – original draft (equal). **Robert M. Plowes:** conceptualization (equal), funding acquisition (lead), methodology (equal), project administration (lead), resources (supporting), supervision (supporting), writing – review and editing (supporting). **Colin R. Morrison:** data curation (supporting), formal analysis (supporting), methodology (supporting), visualization (supporting), writing – review and editing (equal). **Amelia A. Wolf:** conceptualization (equal), resources (equal), writing – review and editing (supporting). **Dino J. Martins:** conceptualization (equal), funding acquisition (supporting), project administration (supporting), resources (equal), supervision (supporting). **Lawrence E. Gilbert:** conceptualization (equal), funding acquisition (lead), project administration (equal), resources (equal), writing – review and editing (supporting).

## Funding

This work was supported by The Lee and Ramona Bass Foundation.

## Conflicts of Interest

The authors declare no conflicts of interest.

## Supporting information


**Figure S1:** CCA denoting the herbaceous plant species in 2024. Ellipses demonstrate groupings by fencing (dotted vs. solid lines) and precipitation regime in color. Species labels were jittered slightly to improve readability while preserving relative positions in ordination space.
**Figure S2:** CCA denoting the woody plant species in 2024. Ellipses demonstrate groupings by fencing (dotted vs. solid lines) and precipitation regime in color. Species labels were jittered to improve readability while preserving relative positions in ordination space.
**Table S1:** Summary of the woody plant species used in the long‐term ungulate exclusion (2024) and rewilding (2025) CCA ordination analyses across fencing treatments (fenced, unfenced, and rewilded) and the six study sites and associated precipitation regime classifications (hydric, xeric, mesic).
**Table S2:** Summary of the herbaceous plant species used in the long‐term ungulate exclusion (2024) and rewilding (2025) CCA ordination analyses across fencing treatments (fenced, unfenced, and rewilded) and the six study sites and associated precipitation regime classifications (hydric, xeric, mesic).
**Table S3:** Summary of the woody and herbaceous plants removed from the long‐term ungulate exclusion (2024) and rewilding (2025) CCA ordination analyses.

## Data Availability

The data and analyses that support the findings of this study are openly available at https://doi.org/10.5281/zenodo.21536452.
