# Supplementary material for: Ungulate Loss and Rewilding Drive Rapid Changes Across Multiple Trophic Levels in an African Savanna
Source: Ecol Evol. 2026 Nov 13;16(11):e74703. doi: 10.1002/ece3.74703 (PMC13768587; doi:10.1002/ece3.74703)
Supplement: Supplementary file 1 [file ECE3-16-e74703-s001.docx]

**Supplement to:**

**Title:** Ungulate loss and rewilding drive rapid changes across multiple trophic levels in an African savanna

**Authors and affiliations**

Aaron C. Rhodes*^a^, Aimee Gaitho^b^, Robert M. Plowes^a^, Colin R. Morrison^a^, Kathryn E. Tisshaw^a^, Amelia A. Wolf^a^, Dino J. Martins^bc^, Lawrence E. Gilbert^a^.

^a^Department of Integrative Biology and Brackenridge Field Laboratory, The University of Texas at Austin, Austin, TX 78703, United States

^b^Mpala Research Centre, P O Box 555 Nanyuki Kenya

^c^Department of Ecology and Evolution, Stony Brook University, Stony Brook, NY 11794

*Corresponding author. [aaronrhodes@utexas.edu](mailto:aaronrhodes@utexas.edu), Brackenridge Field Laboratory, Room 109, 2907 Lake Austin Blvd, Austin, TX 78703, United States

**Summary**

These supplemental additions include the 2024 CCA analyses of woody and herbaceous plant communities, a nematode introduction, methods, results, and discussion that were removed, photos of arthropods encountered in litter, and species tables of those found in the plots for the CCA analyses.

*Woody Plant Community CCA 2024*

The model included canopy cover data of 9 woody plant species associated with fencing and precipitation regime, resulting in three constrained axes. The total inertia of the species data was 6.81, of which 0.69 (10%) was explained by the constrained component, while 6.12 (90%) remained unconstrained. A significant interaction between precipitation and fencing (F = 5.4, p = 0.001), indicated that the effect of fencing on community composition varied across precipitation regimes. Precipitation regime explained the largest portion of constrained variation (F = 32, p = 0.001), followed by fencing (F = 20, p = 0.001).

Species cluster along the primary canonical axes (Fig. S2), indicating that both precipitation regime and fencing treatment contributed to differences in herbaceous species assemblages in 2024. Specifically, *Euclea divinorum*, *Grewia tenax*, *Acacia gerardii*, and *Croton dichogamus* canopy cover was higher in hydric sites, with only the latter higher in unfenced than fenced sites. In mesic and xeric sites, *Acacia brevispica* and *Commiphora africana* canopy cover was higher in fenced sites compared to sites with ungulate herbivory.

Figure S2 - CCA denoting the woody plant species in 2024. Ellipses demonstrate groupings by fencing (dotted vs solid lines) and precipitation regime in color. Species labels were jittered to improve readability while preserving relative positions in ordination space.

*Herbaceous 2024*

The model included cover data for 62 herbaceous plant species and two categorical explanatory variables (fencing and precipitation regime), resulting in three constrained axes. The total inertia of the species data was 6.1, of which 0.70 (10%) was explained by the constrained component, while 6.2 (90%) remained unconstrained. The third axis did not provide additional significant information (p = 1.0). A significant interaction between fencing and precipitation (F *=* 1.29, p = 0.012) indicated that the effect of fencing on community composition varied across precipitation regimes. Precipitation regime explained the largest portion of constrained variation (F *=* 2.92, p = 0.001), followed by fencing (F *=* 1.89, p = 0.001). The first two canonical axes were statistically significant (CCA1: F = 3.5, p = 0.001; CCA2: F = 2.6, p = 0.001) and accounted for 79.3% of the constrained variation.

Clustering of species by treatment category is observed along the primary canonical axes (Fig. S1), indicating that both precipitation regime and fencing contributed to differences in herbaceous species assemblages in 2024. Specifically, *Cenchrus ciliaris*, *Justicia matammensis*, *Crotalaria pycnostachya*, *Cynodon plectostachyus*, and *Indigofera schimperi* were the least influenced by the constrained predictor gradients, unaffected by fencing and precipitation regime. Regardless of precipitation regime, *Ocimum gratissimum*, *Portulaca foliosa*, *Sporobolus festivus*, and *Microchloa kunthii* were more common when ungulates were excluded, while *Pentanisia ouranogyne*, *Kyllinga monocephala*, *Melhania ovata*, *Acacia etbaica*, and *Hibiscus trionum* were more common in unfenced plots. *Eragrostis superba*, *Aristida kenyensis*, *Aristida adoensis*, *Barleria trispinosa*, and *Bothriochloa radicans* were more common in hydric sites, regardless of fencing treatment. *Portulaca oleracea* was more common at xeric sites, regardless of fencing. In xeric sites, no patterns across fencing were seen, while *Ipomoea sinensis*, *Asparagus africanus*, *Eragrostis namaquensis*, and *Oxygonum sinuatum* were more common in the presence of ungulate herbivory. *Brachiaria leersoides* and *Plectranthus caninus* were more common in mesic habitats, regardless of fencing. In mesic sites, no patterns of plants were found outside of fencing, while *Hibiscus micranthus*, *Portulaca quadrifida*, and *Ruellia patula* were more common in the fencing treatment.

Figure S1 – CCA denoting the herbaceous plant species in 2024. Ellipses demonstrate groupings by fencing (dotted vs solid lines) and precipitation regime in color. Species labels were jittered slightly to improve readability while preserving relative positions in ordination space.

*Nematode analyses*

Nematode diversity is shaped not only by ecological factors but also by edaphic characteristics, which influence community structure and abundance (Van Den Hoogen et al. 2019, Nisa et al. 2021). Nematodes are increasingly used as indicators of soil health (Lu et al. 2020), given their fundamental roles in soil nutrient cycling, decomposition, and food webs, and can impact the soil environment similarly to vertebrates in some ecosystems (Ingham and Detling 1986). Ungulates alter soil physical and chemical processes, exerting top-down effects on soil fauna (Ramirez et al. 2021), which can negatively affect root-feeding nematodes (Sitters and Andriuzzi 2019). Nematode diversity serves as a proxy for belowground community diversity (Wubs et al. 2019) and serves important ecological roles as beneficial decomposers and nutrient cyclers; they can also be predatory or parasitic on plants and animals. While ungulates alter soil properties, and nematode diversity is linked to these properties (Nisa et al. 2021), it is unclear how ungulates affect nematode functional diversity.

Plant-parasitic nematodes were detected at low, patchy densities (0–30 individuals per 100 ml soil-1) across treatments and years, with no significant differences among ungulate-exclusion, rewilding, and control plots. Plant-parasitic nematodes were generally rare and occurred at low densities across sites. Total plant-parasitic counts (*Tylenchorhynchus*, *Tylenchulus*, *Hemicycliophora*, *Tylenchus*) ranged from 0 to 30 individuals per 100 ml of soil, with non-zero values in only 11 of 18 samples. The highest density was observed in a single fenced plot (30 individuals per 100 ml), while all other positive samples contained 5–15 individuals per 100 ml.

Indirect impacts on nematodes were inconclusive, despite nematode diversity being highly dependent on soil physical and chemical properties (Nisa et al. 2021), which ungulates are known to alter (Hobbs 1996). Nematode communities in the buffelgrass rhizosphere showed low densities across all treatments, with no significant differences among fenced, rewilded, and unfenced plots. These organisms are found across soil surfaces worldwide, with densities typically lower in tropical zones (Van Den Hoogen et al. 2019), suggesting that an association with ungulates is more important in temperate or Arctic regions (Ramirez et al. 2021). Despite changes in litter depth and soil compaction, these indirect effects did not cause shifts in nematode abundance or composition, contrasting with more responsive aboveground arthropods. This lack of response resets our expectations that ungulate exclusion would alter nematode communities via reduced compaction, as compaction alone halved penetration depth without affecting soil biota here. Low detection may reflect methodological limits of Baermann-funnel extraction for low-density populations or the low abundances in unmanaged tropical ecosystems (Van Den Hoogen et al. 2019, Li et al. 2022). Rewilding failed to alter nematode patterns, despite rapid recovery of litter and compaction over 9 months. Despite the significant impact nematodes have on ecology and agronomy, they remain understudied, with a small proportion of species described (Háněl 1993). We advocate for a broader study of nematode communities at larger spatial and temporal scales. Future work should target molecular methods to resolve free-living vs. parasitic guilds, informing whether ungulate loss truly spares soil food webs or merely obscures subtle shifts.

Supplemental Arthopod images:

Table S1: Summary of the woody plant species used in the long-term ungulate exclusion (2024) and rewilding (2025) CCA ordination analyses across fencing treatments (fenced, unfenced, and rewilded) and the six study sites and associated precipitation regime classifications (hydric, xeric, mesic).

| **Species** | **Year** | **Precipitation regime** | **Site** | **Fencing** |
| --- | --- | --- | --- | --- |
| **Acbr (*Acacia brevispica*)** | 2024 | Hydric | Lookout | Fenced |
|  |  |  | Smithsonian | Fenced, Unfenced |
|  |  | Mesic | Clifford | Fenced |
|  |  |  | Mukenye | Fenced, Unfenced |
|  |  | Xeric | Daraboi | Fenced, Unfenced |
|  |  |  | North Tower | Fenced |
|  | 2025 | Hydric | Lookout | Fenced, Rewilded, Unfenced |
|  |  |  | Smithsonian | Fenced, Rewilded, Unfenced |
|  |  | Mesic | Clifford | Fenced, Rewilded |
|  |  |  | Mukenye | Fenced, Rewilded, Unfenced |
|  |  | Xeric | Daraboi | Fenced, Rewilded, Unfenced |
|  |  |  | North Tower | Rewilded |
| **Acet (*Acacia etbaica*)** | 2024 | Hydric | Lookout | Fenced, Unfenced |
|  |  | Mesic | Clifford | Fenced, Unfenced |
|  |  |  | Mukenye | Fenced, Unfenced |
|  |  | Xeric | Daraboi | Fenced, Unfenced |
|  |  |  | North Tower | Fenced, Unfenced |
|  | 2025 | Hydric | Lookout | Fenced, Rewilded, Unfenced |
|  |  | Mesic | Clifford | Fenced, Rewilded, Unfenced |
|  |  |  | Mukenye | Fenced, Rewilded, Unfenced |
|  |  | Xeric | Daraboi | Fenced, Rewilded, Unfenced |
|  |  |  | North Tower | Fenced, Unfenced |
| **Acge (*Acacia gerardii*)** | 2024 | Hydric | Smithsonian | Fenced, Unfenced |
|  | 2025 | Hydric | Smithsonian | Fenced, Rewilded, Unfenced |
| **Acme (*Acacia mellifera*)** | 2024 | Hydric | Lookout | Fenced |
|  |  |  | Smithsonian | Fenced, Unfenced |
|  |  | Mesic | Clifford | Fenced, Unfenced |
|  |  |  | Mukenye | Fenced, Unfenced |
|  |  | Xeric | Daraboi | Fenced, Unfenced |
|  |  |  | North Tower | Fenced, Unfenced |
|  | 2025 | Hydric | Lookout | Fenced, Rewilded |
|  |  |  | Smithsonian | Fenced, Rewilded, Unfenced |
|  |  | Mesic | Clifford | Fenced, Rewilded, Unfenced |
|  |  |  | Mukenye | Fenced, Rewilded, Unfenced |
|  |  | Xeric | Daraboi | Fenced, Rewilded, Unfenced |
|  |  |  | North Tower | Fenced, Rewilded, Unfenced |
| **Baae (*Balanites aegyptiaca*)** | 2024 | Hydric | Lookout | Fenced, Unfenced |
|  |  |  | Smithsonian | Fenced, Unfenced |
|  |  | Mesic | Clifford | Fenced, Unfenced |
|  |  |  | Mukenye | Fenced |
|  |  | Xeric | Daraboi | Unfenced |
|  |  |  | North Tower | Unfenced |
|  | 2025 | Hydric | Lookout | Fenced, Rewilded, Unfenced |
|  |  |  | Smithsonian | Fenced, Rewilded, Unfenced |
|  |  | Mesic | Clifford | Fenced, Rewilded, Unfenced |
|  |  |  | Mukenye | Fenced |
|  |  | Xeric | Daraboi | Unfenced |
|  |  |  | North Tower | Unfenced |
| **Coaf (*Commiphora africana*)** | 2024 | Hydric | Lookout | Fenced |
|  |  | Mesic | Clifford | Fenced |
|  |  | Mesic | Mukenye | Fenced |
|  |  | Xeric | Daraboi | Fenced, Unfenced |
| **Crdi (*Croton dichogamus*)** | 2024 | Hydric | Lookout | Fenced, Unfenced |
|  |  |  | Smithsonian | Fenced, Unfenced |
|  |  | Mesic | Clifford | Fenced, Unfenced |
|  |  |  | Mukenye | Fenced, Unfenced |
|  |  | Xeric | North Tower | Fenced, Unfenced |
|  | 2025 | Hydric | Lookout | Fenced, Rewilded, Unfenced |
|  |  |  | Smithsonian | Fenced, Rewilded, Unfenced |
|  |  | Mesic | Clifford | Fenced, Rewilded, Unfenced |
|  |  |  | Mukenye | Fenced, Rewilded, Unfenced |
|  |  | Xeric | North Tower | Rewilded, Unfenced |
| **Eudi (*Euclea divinorum*)** | 2024 | Hydric | Lookout | Fenced |
|  |  |  | Smithsonian | Fenced, Unfenced |
|  | 2025 | Hydric | Lookout | Rewilded |
|  |  |  | Smithsonian | Fenced, Rewilded, Unfenced |
| **Eubu (*Euphorbia bussei*)** | 2025 | Xeric | Daraboi | Fenced, Rewilded, Unfenced |
|  |  |  | North Tower | Fenced, Rewilded, Unfenced |
| **Grte (*Grewia tenax*)** | 2024 | Hydric | Lookout | Fenced, Unfenced |
|  |  |  | Smithsonian | Fenced, Unfenced |
|  |  | Mesic | Clifford | Fenced |
|  |  |  | Mukenye | Fenced |
|  |  | Xeric | Daraboi | Fenced |
|  | 2025 | Hydric | Lookout | Fenced, Rewilded, Unfenced |
|  |  |  | Smithsonian | Rewilded, Unfenced |
|  |  | Mesic | Clifford | Fenced, Rewilded |
|  |  |  | Mukenye | Fenced, Rewilded |
|  |  | Xeric | Daraboi | Fenced, Rewilded |

Table S2: Summary of the herbaceous plant species used in the long-term ungulate exclusion (2024) and rewilding (2025) CCA ordination analyses across fencing treatments (fenced, unfenced, and rewilded) and the six study sites and associated precipitation regime classifications (hydric, xeric, mesic).

| **Species** | **Year** | **Precipitation regime** | **Site** | **Fencing** |
| --- | --- | --- | --- | --- |
| **Acet (*Acacia etbaica*)** | 2024 | Hydric | Lookout | Fenced, Unfenced |
|  |  | Xeric | Daraboi | Unfenced |
|  |  |  | North Tower | Unfenced |
|  | 2025 | Hydric | Lookout | Fenced |
|  |  | Xeric | North Tower | Unfenced |
| **Arad (*Aristida adoensis*)** | 2024 | Hydric | Lookout | Fenced, Unfenced |
|  | 2025 | Hydric | Lookout | Rewilded, Unfenced |
| **Arke (*Aristida kenyensis*)** | 2024 | Hydric | Lookout | Fenced, Unfenced |
|  |  |  | Smithsonian | Fenced |
|  | 2025 | Hydric | Lookout | Fenced, Unfenced |
|  |  |  | Smithsonian | Fenced |
|  |  | Mesic | Clifford | Unfenced |
|  |  |  | Mukenye | Rewilded |
| **Asaf (*Asparagus africanus*)** | 2024 | Xeric | Daraboi | Fenced, Unfenced |
|  | 2025 | Xeric | Daraboi | Fenced, Unfenced |
|  |  |  | North Tower | Rewilded |
| **Baac (*Barleria acanthoides*)** | 2024 | Mesic | Clifford | Fenced, Unfenced |
|  |  | Xeric | North Tower | Fenced |
|  | 2025 | Hydric | Smithsonian | Rewilded |
|  |  | Xeric | North Tower | Fenced, Rewilded, Unfenced |
| **Batr (*Barleria trispinosa*)** | 2024 | Hydric | Smithsonian | Fenced, Unfenced |
|  | 2025 | Mesic | Clifford | Fenced, Unfenced |
| **Bora (*Bothriochloa radicans*)** | 2024 | Hydric | Smithsonian | Fenced, Unfenced |
|  | 2025 | Hydric | Smithsonian | Fenced, Rewilded, Unfenced |
| **Brle (*Brachiaria leersoides*)** | 2024 | Mesic | Clifford | Fenced |
| **Ceci (*Cenchrus ciliaris*)** | 2024 | Hydric | Lookout | Fenced, Unfenced |
|  |  |  | Smithsonian | Fenced, Unfenced |
|  |  | Mesic | Clifford | Fenced, Unfenced |
|  |  |  | Mukenye | Fenced, Unfenced |
|  |  | Xeric | Daraboi | Fenced, Unfenced |
|  |  |  | North Tower | Fenced, Unfenced |
|  | 2025 | Hydric | Lookout | Fenced, Rewilded, Unfenced |
|  |  |  | Smithsonian | Fenced, Rewilded, Unfenced |
|  |  | Mesic | Clifford | Fenced, Rewilded, Unfenced |
|  |  |  | Mukenye | Fenced, Rewilded, Unfenced |
|  |  | Xeric | Daraboi | Fenced, Rewilded, Unfenced |
|  |  |  | North Tower | Fenced, Rewilded, Unfenced |
| **Chro (*Chloris roxburghiana*)** | 2024 | Mesic | Clifford | Fenced, Unfenced |
|  |  |  | Mukenye | Fenced, Unfenced |
|  |  | Xeric | Daraboi | Fenced, Unfenced |
|  |  |  | North Tower | Fenced |
|  | 2025 | Mesic | Clifford | Fenced, Unfenced |
|  |  |  | Mukenye | Fenced, Rewilded, Unfenced |
|  |  | Xeric | Daraboi | Fenced, Unfenced |
|  |  |  | North Tower | Fenced |
| **Chvi (*Chloris virgata*)** | 2024 | Mesic | Clifford | Fenced, Unfenced |
|  |  | Xeric | Daraboi | Unfenced |
|  |  |  | North Tower | Fenced |
| **Clmo (*Cleome monophylla*)** | 2024 | Mesic | Mukenye | Fenced |
|  |  | Xeric | North Tower | Fenced |
| **Cola (*Commelina latifolia*)** | 2024 | Hydric | Lookout | Fenced, Unfenced |
|  |  | Mesic | Clifford | Fenced, Unfenced |
|  |  |  | Mukenye | Fenced |
|  |  | Xeric | Daraboi | Fenced, Unfenced |
|  |  |  | North Tower | Fenced, Unfenced |
|  | 2025 | Hydric | Lookout | Fenced, Rewilded, Unfenced |
|  |  | Mesic | Clifford | Rewilded, Unfenced |
|  |  |  | Mukenye | Fenced, Rewilded |
|  |  | Xeric | Daraboi | Fenced, Rewilded, Unfenced |
|  |  |  | North Tower | Fenced, Rewilded |
| **Cost (*Conyza stricta*)** | 2024 | Hydric | Lookout | Fenced, Unfenced |
|  |  | Mesic | Clifford | Unfenced |
|  | 2025 | Hydric | Lookout | Fenced |
|  |  | Mesic | Mukenye | Fenced |
| **Crpl (*Craterostigma plantagineum*)** | 2024 | Hydric | Lookout | Fenced |
| **Crpy (*Crotalaria pycnostachya*)** | 2024 | Hydric | Lookout | Fenced |
|  |  |  | Smithsonian | Fenced |
|  |  | Mesic | Mukenye | Fenced, Unfenced |
|  |  | Xeric | Daraboi | Fenced, Unfenced |
|  |  |  | North Tower | Fenced, Unfenced |
|  | 2025 | Hydric | Lookout | Rewilded |
|  |  | Xeric | Daraboi | Rewilded |
| **Cycy (*Cyathula cylindrica*)** | 2024 | Mesic | Clifford | Fenced, Unfenced |
| **Cyma (*Cyperus mariscus*)** | 2025 | Hydric | Lookout | Unfenced |
| **Cypl (*Cynodon plectostachyus*)** | 2024 | Hydric | Lookout | Fenced, Unfenced |
|  |  | Mesic | Clifford | Fenced, Unfenced |
|  |  |  | Mukenye | Fenced, Unfenced |
|  |  | Xeric | Daraboi | Fenced, Unfenced |
|  |  |  | North Tower | Fenced, Unfenced |
|  | 2025 | Hydric | Lookout | Fenced, Rewilded, Unfenced |
|  |  | Mesic | Clifford | Fenced, Rewilded, Unfenced |
|  |  |  | Mukenye | Fenced, Rewilded, Unfenced |
|  |  | Xeric | Daraboi | Fenced, Rewilded, Unfenced |
|  |  |  | North Tower | Fenced, Rewilded, Unfenced |
| **Cypo (*Cymbopogon pospischilii*)** | 2025 | Mesic | Mukenye | Rewilded, Unfenced |
| **Cyun (*Cyathula uncinulata*)** | 2024 | Mesic | Mukenye | Fenced, Unfenced |
|  |  | Xeric | North Tower | Fenced |
|  | 2025 | Mesic | Mukenye | Unfenced |
|  |  | Xeric | North Tower | Fenced, Unfenced |
| **Emsp (*Emilia* sp.)** | 2024 | Hydric | Lookout | Unfenced |
|  |  | Mesic | Mukenye | Fenced |
|  |  | Xeric | North Tower | Fenced |
| **Erna (*Eragrostis namaquensis*)** | 2024 | Mesic | Clifford | Unfenced |
|  |  | Xeric | Daraboi | Unfenced |
|  |  |  | North Tower | Fenced, Unfenced |
| **Erri (*Eragrostis rigidior*)** | 2025 | Mesic | Clifford | Unfenced |
|  |  | Xeric | Daraboi | Unfenced |
| **Ersu (*Eragrostis superba*)** | 2024 | Hydric | Lookout | Fenced, Unfenced |
|  | 2025 | Hydric | Lookout | Rewilded, Unfenced |
| **Erte (*Eragrostis tenuifolia*)** | 2024 | Hydric | Lookout | Unfenced |
|  |  |  | Smithsonian | Unfenced |
|  |  | Mesic | Clifford | Unfenced |
|  |  | Xeric | Daraboi | Fenced, Unfenced |
|  |  |  | North Tower | Unfenced |
|  | 2025 | Hydric | Lookout | Unfenced |
|  |  |  | Smithsonian | Unfenced |
| **Euhi (*Euphorbia hirta*)** | 2024 | Mesic | Clifford | Unfenced |
|  |  | Xeric | Daraboi | Fenced |
| **Euin (*Euphorbia inaquilatera*)** | 2024 | Xeric | Daraboi | Fenced |
| **Eusc (*Euphorbia scarlatina*)** | 2025 | Xeric | Daraboi | Fenced, Rewilded, Unfenced |
| **Eval (*Evolvulus alsinoides*)** | 2024 | Hydric | Smithsonian | Fenced, Unfenced |
|  |  | Xeric | Daraboi | Unfenced |
|  | 2025 | Xeric | Daraboi | Unfenced |
|  |  |  | North Tower | Unfenced |
| **Guco (*Gutenbergia cordifolia*)** | 2024 | Xeric | Daraboi | Fenced, Unfenced |
|  |  |  | North Tower | Fenced, Unfenced |
|  | 2025 | Xeric | Daraboi | Rewilded, Unfenced |
|  |  |  | North Tower | Fenced, Rewilded, Unfenced |
| **Hasc (*Harpachne schimperi*)** | 2024 | Hydric | Lookout | Fenced, Unfenced |
|  |  | Xeric | Daraboi | Unfenced |
| **Heco (*Heteropogon contortus*)** | 2024 | Hydric | Smithsonian | Fenced, Unfenced |
|  |  | Mesic | Mukenye | Fenced, Unfenced |
|  |  | Xeric | Daraboi | Fenced, Unfenced |
|  | 2025 | Hydric | Lookout | Rewilded |
|  |  |  | Smithsonian | Rewilded, Unfenced |
|  |  | Mesic | Mukenye | Fenced, Rewilded, Unfenced |
|  |  | Xeric | Daraboi | Unfenced |
| **Hegl (*Helichrysum glumaceum*)** | 2024 | Hydric | Lookout | Unfenced |
|  |  |  | Smithsonian | Fenced, Unfenced |
|  | 2025 | Hydric | Lookout | Unfenced |
|  |  |  | Smithsonian | Rewilded, Unfenced |
| **Hime (*Hibiscus meyeri*)** | 2024 | Hydric | Lookout | Unfenced |
|  |  | Mesic | Clifford | Fenced, Unfenced |
|  |  |  | Mukenye | Fenced, Unfenced |
|  |  | Xeric | Daraboi | Fenced |
|  |  |  | North Tower | Fenced, Unfenced |
|  | 2025 | Mesic | Clifford | Fenced, Unfenced |
|  |  |  | Mukenye | Fenced, Rewilded, Unfenced |
|  |  | Xeric | Daraboi | Fenced, Rewilded |
|  |  |  | North Tower | Fenced |
| **Himi (*Hibiscus micranthus*)** | 2024 | Hydric | Smithsonian | Fenced |
|  |  | Mesic | Clifford | Fenced, Unfenced |
|  |  |  | Mukenye | Fenced, Unfenced |
|  |  | Xeric | Daraboi | Fenced |
|  | 2025 | Mesic | Clifford | Fenced, Rewilded |
|  |  |  | Mukenye | Fenced, Unfenced |
| **Hitr (*Hibiscus trionum*)** | 2024 | Hydric | Smithsonian | Unfenced |
|  |  | Xeric | Daraboi | Fenced |
| **Hyfo (*Hypoestes forskaolii*)** | 2024 | Mesic | Mukenye | Unfenced |
|  |  | Xeric | Daraboi | Fenced, Unfenced |
|  |  | Xeric | North Tower | Unfenced |
| **Inar (*Indigofera arrecta*)** | 2024 | Hydric | Lookout | Unfenced |
|  |  |  | Smithsonian | Fenced, Unfenced |
|  |  | Mesic | Clifford | Fenced, Unfenced |
|  |  |  | Mukenye | Fenced, Unfenced |
|  |  | Xeric | Daraboi | Fenced, Unfenced |
|  |  |  | North Tower | Fenced, Unfenced |
|  | 2025 | Hydric | Lookout | Rewilded, Unfenced |
|  |  |  | Smithsonian | Fenced, Unfenced |
|  |  | Mesic | Clifford | Fenced, Rewilded, Unfenced |
|  |  |  | Mukenye | Fenced, Rewilded, Unfenced |
|  |  | Xeric | Daraboi | Rewilded, Unfenced |
|  |  |  | North Tower | Fenced, Rewilded, Unfenced |
| **Insc (*Indigofera schimperi*)** | 2024 | Hydric | Lookout | Fenced |
|  |  | Mesic | Clifford | Unfenced |
|  |  | Xeric | North Tower | Fenced |
| **Ipga (*Ipomoea garckeana*)** | 2024 | Hydric | Lookout | Fenced, Unfenced |
|  |  |  | Smithsonian | Fenced, Unfenced |
|  |  | Mesic | Clifford | Fenced, Unfenced |
|  |  |  | Mukenye | Fenced |
|  |  | Xeric | Daraboi | Fenced, Unfenced |
|  |  |  | North Tower | Fenced, Unfenced |
|  | 2025 | Hydric | Lookout | Unfenced |
|  |  | Mesic | Mukenye | Fenced |
|  |  | Xeric | Daraboi | Fenced |
|  |  |  | North Tower | Rewilded |
| **Ipsi (*Ipomoea sinensis*)** | 2024 | Xeric | North Tower | Fenced, Unfenced |
| **Juma (*Justicia matammensis*)** | 2024 | Hydric | Lookout | Fenced, Unfenced |
|  |  |  | Smithsonian | Fenced |
|  |  | Mesic | Clifford | Fenced, Unfenced |
|  |  |  | Mukenye | Fenced, Unfenced |
|  |  | Xeric | Daraboi | Fenced, Unfenced |
|  |  |  | North Tower | Unfenced |
|  | 2025 | Hydric | Lookout | Fenced, Rewilded, Unfenced |
|  |  | Mesic | Clifford | Fenced, Rewilded, Unfenced |
|  |  |  | Mukenye | Rewilded |
| **Kymo (*Kyllinga monocephala*)** | 2024 | Hydric | Lookout | Unfenced |
|  |  | Mesic | Clifford | Unfenced |
|  |  |  | Mukenye | Unfenced |
|  |  | Xeric | North Tower | Fenced, Unfenced |
| **Lede (*Leucas deflexa*)** | 2024 | Hydric | Lookout | Unfenced |
|  |  |  | Smithsonian | Fenced |
|  |  | Mesic | Clifford | Fenced, Unfenced |
|  |  |  | Mukenye | Fenced |
|  |  | Xeric | North Tower | Unfenced |
| **Meov (*Melhania ovata*)** | 2024 | Hydric | Lookout | Fenced, Unfenced |
|  |  |  | Smithsonian | Fenced, Unfenced |
|  |  | Mesic | Clifford | Fenced, Unfenced |
|  |  |  | Mukenye | Unfenced |
|  |  | Xeric | Daraboi | Unfenced |
|  |  |  | North Tower | Unfenced |
|  | 2025 | Hydric | Lookout | Unfenced |
|  |  |  | Smithsonian | Fenced |
|  |  | Mesic | Clifford | Unfenced |
|  |  |  | Mukenye | Unfenced |
|  |  | Xeric | Daraboi | Rewilded, Unfenced |
|  |  |  | North Tower | Unfenced |
| **Mere (*Melinis repens*)** | 2024 | Mesic | Clifford | Fenced |
|  |  | Xeric | Daraboi | Unfenced |
| **Miku (*Microchloa kunthii*)** | 2024 | Hydric | Lookout | Fenced, Unfenced |
|  |  | Mesic | Clifford | Fenced, Unfenced |
|  |  |  | Mukenye | Fenced, Unfenced |
|  |  | Xeric | Daraboi | Fenced |
|  |  |  | North Tower | Fenced |
|  | 2025 | Hydric | Lookout | Fenced, Unfenced |
|  |  | Mesic | Clifford | Unfenced |
|  |  |  | Mukenye | Rewilded, Unfenced |
|  |  | Xeric | Daraboi | Fenced, Rewilded, Unfenced |
|  |  |  | North Tower | Fenced, Unfenced |
| **Moan (*Monsonia angustifolia*)** | 2024 | Hydric | Lookout | Unfenced |
|  |  |  | Smithsonian | Unfenced |
|  |  | Mesic | Clifford | Unfenced |
|  |  | Xeric | Daraboi | Unfenced |
| **Ocgr (*Ocimum gratissimum*)** | 2024 | Hydric | Lookout | Fenced, Unfenced |
|  |  |  | Smithsonian | Fenced |
|  |  | Mesic | Clifford | Fenced |
|  |  |  | Mukenye | Fenced |
|  |  | Xeric | Daraboi | Fenced |
|  | 2025 | Hydric | Lookout | Rewilded |
|  |  |  | Smithsonian | Fenced |
|  |  | Mesic | Mukenye | Rewilded |
| **Oxsi (*Oxygonum sinuatum*)** | 2024 | Hydric | Lookout | Fenced, Unfenced |
|  |  | Mesic | Mukenye | Fenced |
|  |  | Xeric | Daraboi | Unfenced |
|  |  |  | North Tower | Fenced, Unfenced |
| **Peou (*Pentanisia ouranogyne*)** | 2024 | Hydric | Lookout | Fenced, Unfenced |
|  |  | Xeric | Daraboi | Fenced, Unfenced |
|  |  | Xeric | North Tower | Unfenced |
| **Pest (*Pennisetum stramineum*)** | 2024 | Hydric | Smithsonian | Unfenced |
|  |  | Mesic | Clifford | Unfenced |
|  | 2025 | Hydric | Smithsonian | Unfenced |
|  |  | Mesic | Clifford | Unfenced |
| **Pham (*Phyllanthus amarus*)** | 2024 | Hydric | Lookout | Unfenced |
|  |  |  | Smithsonian | Unfenced |
|  |  | Mesic | Clifford | Fenced, Unfenced |
|  |  |  | Mukenye | Unfenced |
|  |  | Xeric | Daraboi | Unfenced |
|  |  |  | North Tower | Unfenced |
| **Plca (*Plectranthus caninus*)** | 2024 | Mesic | Clifford | Fenced |
|  | 2025 | Mesic | Clifford | Fenced |
|  |  |  | Clifford | Rewilded |
| **Pofo (*Portulaca foliosa*)** | 2024 | Hydric | Lookout | Fenced, Unfenced |
|  |  | Mesic | Clifford | Fenced |
|  | 2025 | Hydric | Lookout | Fenced, Unfenced |
|  |  | Mesic | Clifford | Rewilded |
|  |  |  | Mukenye | Unfenced |
|  |  | Xeric | North Tower | Fenced, Rewilded |
| **Pool (*Portulaca oleracea*)** | 2024 | Xeric | Daraboi | Unfenced |
|  |  |  | North Tower | Unfenced |
| **Poqu (*Portulaca quadrifida*)** | 2024 | Mesic | Mukenye | Fenced, Unfenced |
| **Post (*Polygala stenopetala*)** | 2024 | Hydric | Smithsonian | Fenced |
|  |  | Mesic | Clifford | Unfenced |
|  |  |  | Mukenye | Fenced, Unfenced |
|  |  | Xeric | Daraboi | Fenced, Unfenced |
|  | 2025 | Hydric | Lookout | Fenced |
|  |  | Mesic | Mukenye | Rewilded |
|  |  | Xeric | Daraboi | Rewilded |
|  |  |  | North Tower | Fenced, Unfenced |
| **Rupa (*Ruellia patula*)** | 2024 | Hydric | Lookout | Unfenced |
|  |  |  | Smithsonian | Fenced |
|  |  | Mesic | Clifford | Fenced |
|  |  |  | Mukenye | Fenced |
|  |  | Xeric | Daraboi | Unfenced |
|  |  |  | North Tower | Fenced |
|  | 2025 | Hydric | Smithsonian | Fenced |
|  |  | Mesic | Mukenye | Fenced |
|  |  | Xeric | North Tower | Fenced |
| **Rupr (*Ruellia prostrata*)** | 2025 | Mesic | Clifford | Fenced, Rewilded |
| **Sene (*Sehima nervosum*)** | 2025 | Hydric | Smithsonian | Rewilded, Unfenced |
|  |  | Xeric | Daraboi | Unfenced |
| **Siov (*Sida ovata*)** | 2024 | Hydric | Smithsonian | Fenced, Unfenced |
|  | 2025 | Hydric | Smithsonian | Unfenced |
|  |  | Xeric | North Tower | Unfenced |
| **Soin (*Solanum incanum*)** | 2024 | Hydric | Lookout | Fenced, Unfenced |
|  |  | Mesic | Clifford | Fenced, Unfenced |
|  |  |  | Mukenye | Fenced, Unfenced |
|  |  | Xeric | Daraboi | Fenced, Unfenced |
|  |  |  | North Tower | Unfenced |
|  | 2025 | Hydric | Lookout | Unfenced |
|  |  | Mesic | Clifford | Unfenced |
|  |  |  | Mukenye | Fenced, Rewilded, Unfenced |
|  |  | Xeric | Daraboi | Fenced, Rewilded, Unfenced |
|  |  |  | North Tower | Rewilded, Unfenced |
| **Spfe (*Sporobolus festivus*)** | 2024 | Hydric | Lookout | Fenced |
|  |  | Mesic | Clifford | Unfenced |
|  |  |  | Mukenye | Fenced, Unfenced |
|  |  | Xeric | Daraboi | Fenced, Unfenced |
|  | 2025 | Hydric | Lookout | Fenced |
|  |  | Mesic | Clifford | Rewilded, Unfenced |
|  |  |  | Mukenye | Unfenced |
|  |  | Xeric | Daraboi | Rewilded, Unfenced |
|  |  |  | North Tower | Fenced, Rewilded, Unfenced |
| **Tapo (*Talinum portulacifolium*)** | 2024 | Mesic | Clifford | Fenced |
|  |  | Xeric | North Tower | Fenced |
|  | 2025 | Mesic | Clifford | Fenced, Rewilded |
|  |  | Xeric | North Tower | Rewilded |
| **Thtr (*Themeda triandra*)** | 2024 | Mesic | Clifford | Unfenced |
|  |  |  | Mukenye | Fenced, Unfenced |
|  |  | Xeric | Daraboi | Fenced |
|  |  |  | North Tower | Unfenced |
|  | 2025 | Mesic | Mukenye | Fenced |
|  |  | Xeric | Daraboi | Fenced |
| **Trbe (*Tragus berteronianus*)** | 2024 | Hydric | Lookout | Unfenced |
|  |  | Xeric | Daraboi | Unfenced |
|  |  |  | North Tower | Fenced, Unfenced |

Table S3: Summary of the woody and herbaceous plants removed from the long-term ungulate exclusion (2024) and rewilding (2025) CCA ordination analyses.

| **Woody** | |
| --- | --- |
| **2024** | **2025** |
| *Acacia nilotica* | *Acacia nilotica* |
| *Acacia tortilis* | *Boscia angustifolia* |
| *Boscia angustifolia* | *Canthium coromandelicum* |
| *Carissa edulis* | *Carissa edulis* |
| *Grewia bicolor* | *Cordia africana* |
| *Maerue triphylla* | *Grewia megalocarpa* |
| *Psydrax schimperiana* | *Lannea rivae* |
| *Pyrostria phyllanthoidea* | *Maerue triphylla* |
| *Rhus natalensis* | *Psydrax schimperiana* |
| *Teclea nobilis* | *Pyrostria phyllanthoidea* |
|  | *Rhus natalensis* |
|  | *Teclea nobilis* |
|  | *Zanthoxylum chalybeum* |
| **Herbaceous** | |
| **2024** | **2025** |
| *Chloris gayana* |  |
| *Chrysopogon plumulosus* | *Brachiaria leersoides* |
| *Crinum macowani* | *Caralluma dummeri* |
| *Cucumis africanus* | *Croton dichogamus* |
| *Cyperus pennatiformis* | *Enteropogon macrostachyus* |
| *Cymbopogon pospischilii* | *Eragrostis namaquensis* |
| *Dactyloctenium aegyptium* | *Euphorbia hirta* |
| *Enneapogon cenchroides* | *Euphorbia inaquilatera* |
| *Eragrostis rigidior* | *Harpachne schimperi* |
| *Euphorbia scarlatina* | *Hypoestes forskaolii* |
| *Hibiscus calyphyllus* | *Monsonia angustifolia* |
| *Hibiscus flavoroseus* | *Pennisetum mezianum* |
| *Pennisetum mezianum* | *Pentanisia ouranogyne* |
| *Priva curtisae* | *Phyllanthus amarus* |
| *Rhynchosia caribaea* | *Portulaca quadrifida* |
| *Rhinacanthus ndorensis* | *Rhynchosia caribaea* |
